# Genetic toxicity assessment of engineered nanoparticles using a 3D in vitro skin model (EpiDerm™)

**DOI:** 10.1186/s12989-016-0161-5

**Published:** 2016-09-09

**Authors:** John W. Wills, Nicole Hondow, Adam D. Thomas, Katherine E. Chapman, David Fish, Thierry G. Maffeis, Mark W. Penny, Richard A. Brown, Gareth J. S. Jenkins, Andy P. Brown, Paul A. White, Shareen H. Doak

**Affiliations:** 1 Institute of Life Sciences, Swansea University Medical School, Singleton Park, Swansea, SA2 8PP UK; 2 School of Chemical and Process Engineering, University of Leeds, Leeds, LS2 9JT UK; 3 Multi-Disciplinary Nanotechnology Centre, College of Engineering, Singleton Park, Swansea University, Swansea, SA2 8PP UK; 4 Department of Biology, University of Ottawa, 30 Marie-Curie Private, Ottawa, K1N 9B4 ON Canada

**Keywords:** 3D cell culture, Silica, Genotoxicity, Nanotoxicology, Physico-chemical characterisation, Nanoparticles, Reconstructed skin, RSMN, Micronucleus assay, Air-liquid interface

## Abstract

**Background:**

The rapid production and incorporation of engineered nanomaterials into consumer products alongside research suggesting nanomaterials can cause cell death and DNA damage (genotoxicity) makes in vitro assays desirable for nanosafety screening. However, conflicting outcomes are often observed when in vitro and in vivo study results are compared, suggesting more physiologically representative in vitro models are required to minimise reliance on animal testing.

**Method:**

BASF Levasil® silica nanoparticles (16 and 85 nm) were used to adapt the 3D reconstructed skin micronucleus (RSMN) assay for nanomaterials administered topically or into the growth medium. 3D dose-responses were compared to a 2D micronucleus assay using monocultured human B cells (TK6) after standardising dose between 2D / 3D assays by total nanoparticle mass to cell number. Cryogenic vitrification, scanning electron microscopy and dynamic light scattering techniques were applied to characterise in-medium and air-liquid interface exposures. Advanced transmission electron microscopy imaging modes (high angle annular dark field) and X-ray spectrometry were used to define nanoparticle penetration / cellular uptake in the intact 3D models and 2D monocultured cells.

**Results:**

For all 2D exposures, significant (*p* < 0.002) increases in genotoxicity were observed (≥100 μg/mL) alongside cell viability decreases (*p* < 0.015) at doses ≥200 μg/mL (16 nm-SiO_2_) and ≥100 μg/mL (85 nm-SiO_2_). In contrast, 2D-equivalent exposures to the 3D models (≤300 μg/mL) caused no significant DNA damage or impact on cell viability. Further increasing dose to the 3D models led to probable air-liquid interface suffocation. Nanoparticle penetration / cell uptake analysis revealed no exposure to the live cells of the 3D model occurred due to the protective nature of the skin model’s 3D cellular microarchitecture (topical exposures) and confounding barrier effects of the collagen cell attachment layer (in-medium exposures). 2D monocultured cells meanwhile showed extensive internalisation of both silica particles causing (geno)toxicity.

**Conclusions:**

The results establish the importance of tissue microarchitecture in defining nanomaterial exposure, and suggest 3D in vitro models could play a role in bridging the gap between in vitro and in vivo outcomes in nanotoxicology. Robust exposure characterisation and uptake assessment methods (as demonstrated) are essential to interpret nano(geno)toxicity studies successfully.

**Electronic supplementary material:**

The online version of this article (doi:10.1186/s12989-016-0161-5) contains supplementary material, which is available to authorized users.

## Background

The nano-scaling of materials has led to the identification of enhanced mechanical, optical, electrical, catalytic and magnetic properties relative to that of micro-scaled formulations [1–3]. Consequently, nanomaterials increasingly find applications in commercial products as adoption of nanotechnology undergoes rapid global expansion [4]. Human exposure to engineered nanomaterials is therefore already occurring and looks set to rise in the future.

Though it is size that permits desirable functionality, it is also known to facilitate nanomaterial uptake, tissue penetration and systemic distribution in the body [5, 6]. Concern arises as research shows nanomaterials can stimulate inflammatory responses, initiate oxidative stress and cause DNA damage (genotoxicity) and cell death (cytotoxicity) [5, 7–9]. This has necessitated the development of robust test protocols for nanomaterial safety assessment, with a variety of in vitro and in vivo test methodologies examined to date. It has become clear however that assays designed for chemical hazard assessment may be unsuitable or require considerable optimisation for nanoscale test articles [3, 9–11].

Since pathophysiologic effects in vivo are influenced by both toxicokinetics (i.e., distribution, accumulation and clearance), as well as toxicodynamics (i.e., tissue- and/or cell-specific responses); the use of in vitro tools primarily remains restricted to hazard identification [12]. Furthermore, conflicting results are often found when the results of similar in vitro studies or in vitro and in vivo outcomes are compared. This has raised concern regarding the appropriateness of current in vitro assays for nanoscale test articles, and has encouraged continued reliance on in vivo safety testing strategies for nanomaterials [10, 13]. However, to keep pace with the rapid growth of the nanotechnology sector, and societal and economic pressures to replace, reduce or refine the need for animals in consumer product safety assessments, there is a growing need for robust in vitro alternatives [1].

The conflicting outcomes noted between in vitro and in vivo nanosafety studies have been partially attributed to insufficient particle characterisation in the biological matrix of the employed test system, where bio-nano interactions (e.g., serum protein-to-particle binding) and agglomeration processes are known to modulate nanomaterial surface chemistries and the kinetics of dosimetry [14, 15]. Ultimately these processes affect what is presented at the cell surface and the probability that cellular uptake will occur [13, 16]. Consequently, characterising exposure in the biological environment and quantifying cellular uptake have been highlighted as critical factors for successful interpretation and comparison of nanotoxicological assessments in vitro [17, 18].

There is also wider concern, for both chemical compounds and nanomaterial safety assessment perspectives, that assays based on two-dimensional monocultured cells do not represent the three dimensional complexity of in vivo systems. This is thought to constitute a major factor in the over-predictivity associated with in vitro assays (i.e., low specificity), as three-dimensional (3D) cellular microarchitectures in vivo may constitute a barrier to distribution and absorption that is not represented in 2D monocultures [10, 19–23]. It has therefore been suggested that the development of in vitro assays with 3D cellular structures is critical for ‘bridging the gaps’ between in vitro and in vivo outcomes [10].

Despite existing in a variety of different formats, 3D in vitro models share the characteristic that their constituent cells combinatorially establish a 3D microarchitecture. It has been shown that that this 3D structure can influence diverse cellular functionalities including proliferation, differentiation, migration, invasion and cell death [10]. To date, the majority of 3D culture systems applied in nanotoxicology have been ‘spheroid’ models in which a tight ball of cells is established and employed. Quantum dot, gold, iron oxide, carbon nanotube and silica nanoparticle exposures to such spheroidal cultures established from macrophage and liver cells have all shown different outcomes in comparison to 2D monocultures: more specifically, toxicity and tissue penetration have been reported to be reduced, and restricted to the outermost layers of the spheroids [10, 24–27]. It is to be noted however that these studies primarily focused on the potential for nanomaterials to induce cytotoxicity, whilst arguably the potential for carcinogenesis and heritable genetic alterations via DNA damage and mutagenesis could be considered the more concerning outcomes associated with nanomaterial exposures [7].

The 2D in vitro micronucleus (MN) assay is the gold standard test system for the detection of chromosomal damage induced by an exogenous agent, and nano-specific guidance for conducting test article assessments is becoming defined [11, 28, 29]. Despite the establishment of this guidance however, the short-comings of any 2D system for nano assessment are well acknowledged and there is general agreement that the utility of recently-developed 3D alternatives needs to be explored and evaluated [10]. Recently, intensive international efforts have gone into the development of a 3D reconstructed skin micronucleus (RSMN) assay for chemical test articles (chemical-RSMN [30]), which uses the commercially available EpiDerm^TM^ human epidermis model (MatTek Corporation). The development and pre-validation of this 3D assay has been well documented [22, 30–36].

Given that the 7^th^ Amendment to the Cosmetics Directive in Europe now prohibits the testing of cosmetics and cosmetic ingredients in vivo, alternative approaches to assess dermal exposures are critical. Thus, developing the 3D RSMN assay for nanosafety assessment is of significant interest as the health and fitness industry is increasingly using nanomaterials in personal care products, cosmetics and clothing [37]. Furthermore, the EpiDerm™ model has already shown promise for nanosafety applications including the assessment of nanoparticle skin irritation [38, 39] and percutaneous absorption [40].

This investigation used silica nanoparticles, dermal exposure to which is a concern due to their incorporation in adhesives, polishes and varnishes, photocopier toner, as well as their use as food stabilizing agents and as cosmetic additives [37, 41], to determine the utility of the RSMN protocol for nanosafety assessment. The establishment of equivalent 2D/3D nanoparticle doses permitted dose-response comparisons between the developed, 3D ‘nano-RSMN’ method and a ‘traditional’ 2D micronucleus assay, carried out using monocultured human B lymphoblastoid cells (TK6). TK6 cells were chosen for the 2D studies because their use is recommended in the existing Organisation for Economic Co-operation and Development (OECD) micronucleus assay test guideline (i.e., Test Guideline 487); thus, their use for the in vitro MN assay has been extensively validated and internationally-accepted [42]. Comparing the outcomes of a ‘typical’ 2D in vitro micronucleus test conducted according to the OECD test guideline with a ‘new’ version of the assay employing 3D in vitro models was therefore deemed the appropriate starting point for a comparison between 2D and 3D forms of the assay versions. Alongside, cryogenic vitrification, scanning electron microscopy and dynamic light scattering techniques are demonstrated to characterise 2D in-medium and 3D air-liquid interface exposures. Finally, advanced transmission electron imaging modes were used to define nanoparticle penetration in the intact, 3D architecture of the EpiDerm™ tissues and cellular uptake in the 2D monocultures facilitating robust 2D/3D dose-response comparison.

## Results

### Primary nanoparticle physico-chemical characterisation

**Fig. 1:**
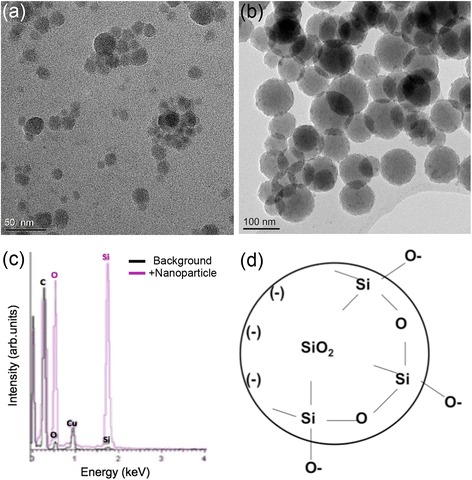
BASF Levasil® silicon dioxide nanoparticle primary characterisation: Bright field TEM micrographs of (**a**) 16 nm-SiO_2_, and (**b**) 85 nm-SiO_2_, allowed primary particle size, shape and morphology to be assessed. **c** Typical particle EDX spectrum relative to background confirming the presence of silicon and oxygen with no detectable contaminants (copper and carbon due to TEM grid and support film). **d** Schematic illustrating the negative surface charge of SiO_2_ particles, due to unbound surface oxygen groups

This study used BASF Levasil® 200 and Levasil® 50 amorphous silica nanoparticles to optimise a 3D RSMN assay for nanomaterial test articles. Transmission electron microscopy (TEM) indicated both particles were spherical and had a relatively smooth surface morphology (Fig. 1a and b). Primary size (i.e., single particle) measurements from electron micrographs determined the average diameter of the Levasil® 200 to be 16.4 nm (manufacturer specified 15 nm) and Levasil® 50 to be 85.1 nm (manufacturer specified 55 nm) (Table 1). Therefore, text references hereafter refer to 16 nm-SiO_2_ or 85 nm-SiO_2_, respectively. No evidence of regular lattice planes was found at higher magnification confirming the expected amorphous structure. Nanoparticle composition and the presence of trace contaminants was investigated using energy dispersive X-ray (EDX) spectrometry. Comparing a blank area of the TEM grid to an area containing nanoparticles revealed a large shift in the ratio of the oxygen and silicon peaks, confirming the silicon dioxide particle composition (Fig. 1c). No evidence of unexpected elemental traces (e.g., impurities or unexpected suspension phase contaminants) was found by EDX. Dynamic Light Scattering (DLS) analysis at 300 μg/mL in ultra-pure water (MilliQ, 18MΩ·cm) showed number distribution peak maxima at 17 nm and 92 nm, respectively, for the 16 nm-SiO_2_ and 85 nm-SiO_2_. DLS size ranges in water were therefore concurrent to the primary sizes established by TEM, suggesting manufacturer-supplied suspensions were colloidally stable. Surface charge (zeta potential) measurements were strongly negative (< −40 mV) for both particles further indicative of colloidal stability in water and consistent with silicon dioxide’s surface chemistry of negative, unbound oxygen groups (Table 1 and Fig. 1d). Further TEM images, particle size distributions and EDX spectra are available in Additional files 1, 2 and 3.

**Table 1:** Levasil® nanoparticle primary characterisation: TEM in conjunction with EDX spectroscopy was used to determine primary size distributions, shape / morphology, crystallinity, composition and purity. DLS determined particle colloidal stability/agglomeration (hydrodynamic diameter) and also particle surface charge (zeta potential) in as-manufactured aqueous solution

Product Name	Text Reference	Manufacturer Specified Size (diameter, nm)	Transmission Electron Microscopy/Energy Dispersive X-ray Spectroscopy			Material Density (g/cm^3^)	Material Refractive Index	Dynamic Light Scattering (peak analysis of distributions by particle number)				
			Primary Size: average diameter, nm ± standard deviation (range)	Shape/Morphology	Crystallinity/Composition/Purity Contaminant free?			Agglomeration: (hydrodynamic diameter, nm)			Surface Charge (mV)	Surface chemistry
								*aqueous dispersion; 300 μg/mL*				
								Modal size (distribution peak max)	Size Range (99 % distribution)	Polydispersity ndex (range)	Zeta Potential ± SD (Solution pH)	
Levasil® 200 (aqueous solution)	16 nm-SiO_2_	15	16.4 ± 5.3 (8 – 42)	spherical/smooth	Amorphous SiO_2_, yes	2.65	1.54	17	9 - 32	0.02 - 0.08	-55.3 ± 2.3 *(pH 7.8)*	negative due to unbound surface oxygen
Levasil® 50 (aqueous solution)	85 nm-SiO_2_	55	85.1 ± 23.7 (41 – 159)	spherical/smooth	Amorphous SiO_2_, yes	2.65	1.54	92	50 - 164	0.21 - 0.22	-63.2 ± 3.8 *(pH 9.4)*	

### Development and optimisation of a reconstructed skin micronucleus (RSMN) assay protocol for nanomaterials (nano-RSMN)

**Fig. 2:**
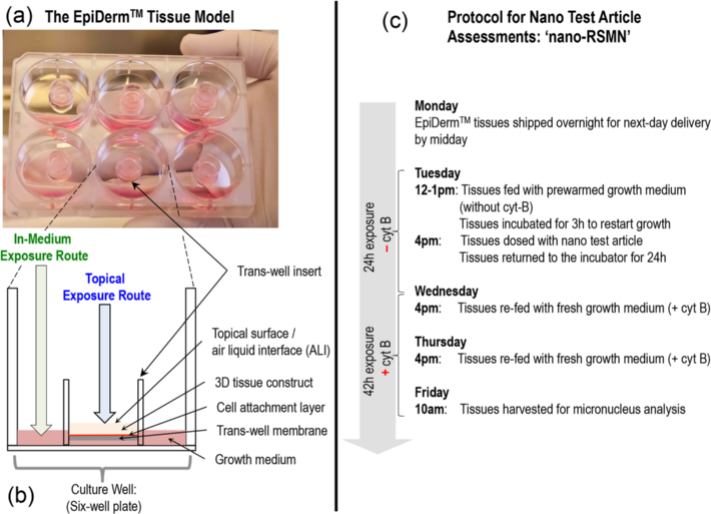
The reconstructed skin micronucleus assay optimised for nanomaterial test articles: (**a**) Six-well plate containing MatTek Corporation’s 3D epidermis (EpiDerm^TM^) tissue models in trans-well inserts. **b** Schematic diagram of a single well (cross-section) highlighting the two nanoparticle exposure routes utilised with the 3D models in this study: nanoparticles were either inoculated onto the topical surface or administered into the growth medium. **c** The developed day-by-day ‘nano-RSMN’ assay protocol from receipt of tissues to harvest detailing dosing, media changes and sequential cyt B regime

Two exposure routes for the 3D skin models were considered in this study: nanoparticles were either applied topically onto the surface of the stratum corneum, mimicking dermal deposition, or were administered directly into the growth medium (in-medium), simulating circulatory exposure (Fig. 2a and b). Maintenance of an air-liquid interface (ALI) at the dermal surface of the model is known to be essential to model viability. The previously published chemical-RSMN method [30] therefore recommends the use of 10 μL acetone, which is commonly used for in vivo dermal exposures, as a delivery vehicle because it quickly evaporates, leaving a dry surface to maintain the ALI. For this reason, and because it was possible to prepare stable colloidal suspensions, the silica nanoparticles used here were also administered in this way. The acetone evaporated in 15–20 s, depositing the particles directly onto the tissue surface.

One of the most important considerations in the MN assay is ensuring that cells complete mitosis during the exposure period, as this is required for lagging chromosomes or chromosomal fragments (i.e. resulting from exposure) to manifest as scorable MN events. Identification of cells that have undergone division is most commonly achieved using cytochalasin B (cyt B) to block cytokinesis, leading to the formation of readily identifiable binucleated cells [42]. However, cyt B acts through actin inhibition and it can inhibit endocytosis processes known to be essential for the active cellular uptake of many nanomaterials. It is therefore important to modify chemical MN assay methodologies to permit a period of nanomaterial exposure in absence of cyt B prior to its sequential addition [11, 28, 29, 42]. For this reason, and in order to present a test methodology that was as comparable as possible to the 24 h exposure-then-recovery regime (i.e., a *sequential* cyt B regime) employed for the 2D MN assay, the ‘nano-RSMN’ method shown in Fig. 2c was developed to permit enumeration of induced, cytokinesis-blocked MNs in the 3D system. The protocol uses a single, 24 h test article exposure (– cyt B) followed by 42 h recovery phase (+ cyt B) to allow the primary cells of the 3D model time to divide.

**Fig. 3:**
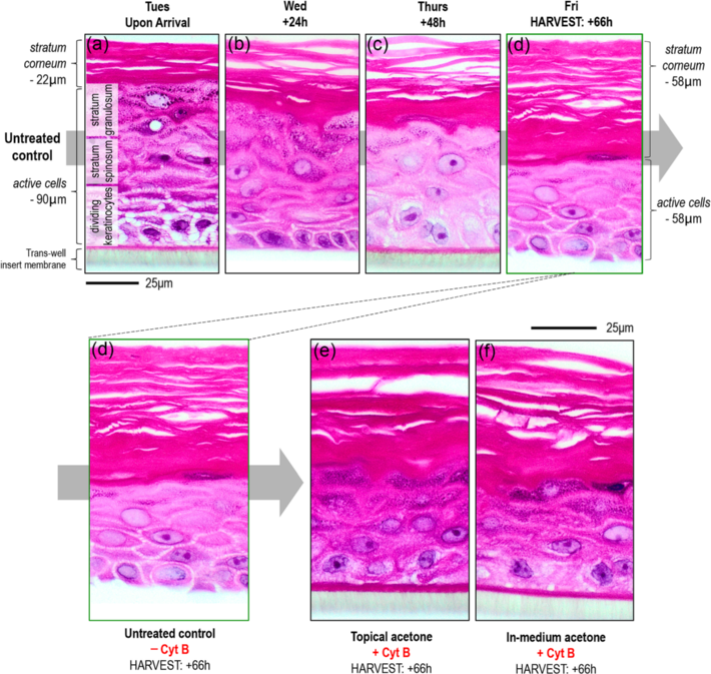
3D model structure, growth and differentiation across the nano-RSMN protocol: The EpiDerm^TM^ model is a structurally differentiated, multi-layered model (**a**) of the human epidermis created from primary human keratinocytes. From arrival (**a**) to harvest (**d**) untreated control tissues in absence of cyt B were grown and imaged daily. The stratum corneum barrier layer increased in thickness as dividing keratinocytes moved upwards and differentiated. (Note: the trans-well insert layer detached during image preparation, (**a**, **d**). Comparison of the untreated control tissue (**d**) to tissues exposed to cyt B and acetone via the topical (**e**) and medium (**f**) exposure routes at the harvest time-point showed no differences in tissue development/structure

To study the impact of cyt B and acetone on tissue growth and differentiation, unexposed control tissues were harvested on each day of the assay, stained with haemotoxylin and eosin and optically imaged in cross-section (Fig. 3a–d). From arrival to harvest, the stratum corneum layer was seen to increase in thickness (from ~22 μm to 58 μm) as dividing basal cells moved upwards and differentiated to form new stratum corneum. The multi-layered structure of the model was revealed with distinct basal cell, stratum spinosum, granulosum and corneum layers identifiable (Fig. 3a). Negative control tissues at the harvest time-point were then compared to tissues dosed with 10 μL topical or in-medium acetone and cultured using cyt B according to the defined nano-RSMN protocol (Fig. 2c). Encouragingly, no difference in tissue structure or morphology caused by solvent or cyt B inclusion was identified. Harvest time-point stratum corneum and basal cell thicknesses remained highly similar to untreated control (Fig. 3d– f).

Despite differences in study duration, number of acetone (dosing) exposures and use of a sequential/shorter exposure cyt B regime, the developed nano-RSMN method also encouragingly yielded comparable binucleated cell frequencies (*p* > 0.17, *n* = 3) to those obtained using the original chemical-RSMN [30] approach. The binucleate frequency data from these optimisation experiments are presented in Additional file 4.

### Dosing and characterising nanomaterial exposures in the 2D and 3D assays

As topical silica nanoparticle exposures were evaporation-deposited onto the tissue surface, the mass/volume (e.g., μg/mL) 2D dosing approach could not be applied directly when dosing the 3D models. Dose between 2D and 3D assays was therefore normalised in terms of total nanoparticle mass to the total number of cells in each assay (see Methods). In this way, ‘3D equivalent’ total mass doses of 150 μg, 300 μg and 450 μg were established to allow comparison to the 100 μg/mL, 200 μg /mL and 300 μg /mL exposures used in the 2D cytotoxicity (relative population doubling (RPD)) and MN assays. Alternative dose metrics including the 3D equivalent total mass doses per unit area (topical exposures) and per unit volume (in-medium exposures) are provided in Table 2.

**Table 2:** 2D/3D dose normalisation and alternative metrics: Dose between 2D and 3D in vitro test systems was standardised in terms of total nanoparticle mass to total cell number at time of dosing (Table Left) (see Methods). For each exposure route used here, alternative dose-metrics (as applicable) are also presented (Table Right) to facilitate comparison. In-medium exposures (2D/3D In-Medium) are provided in total mass (μg), mass/volume (μg/mL) and number/volume (nM) units. Topical, acetone-deposited exposures (3D Topical) are presented in total mass (μg) and mass/surface area (μg/cm^2^) units

This study’s 2D/3D equivalent* exposures:			Alternative dose metrics for each exposure route: (as applicable)						
2D In-Medium (μg/mL)	3D In-Medium (μg)	3D Topical (μg)	2D In-Medium (μg)	3D In-Medium (μg/mL)	3D Topical (μg/cm^2^)	3D In-Medium (nM)		2D In-Medium (nM)	
						16 nm-SiO_2_	85 nm-SiO_2_	16 nm-SiO_2_	85 nm-SiO_2_
0	0	0	0.0	0.0	0.0	0.0	0.00	0.0	0.00
100	150	150	1000.0	167.8	235.9	49.0	0.33	29.2	0.19
200	300	300	2000.0	336.7	473.4	98.4	0.66	58.4	0.39
300	450	450	3000.0	505.6	710.9	147.7	0.99	87.7	0.58
−	1000	1000	−	1111.1	1562.5	324.6	2.17	−	−

*Total nanoparticle mass normalised to number of cells per 2D/3D in vitro system (see methods)

**Fig. 4:**
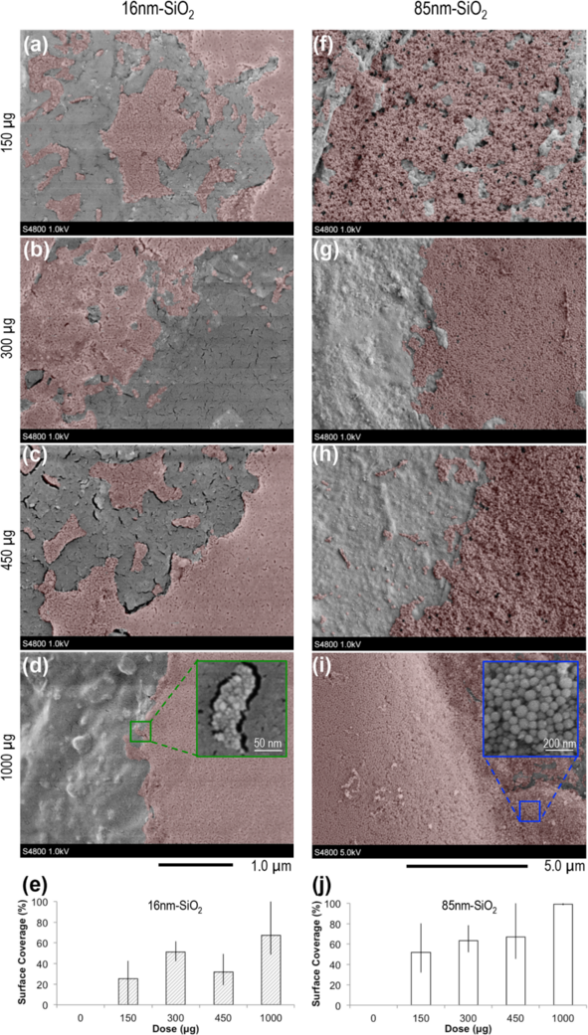
Characterising 3D topical nanosilica exposures after deposition in acetone using cryogenic vitrification and scanning electron microscopy: Representative images 16 nm-SiO_2_ (**a** – **d**), 85 nm-SiO_2_ (**f** – **i**). Particle deposition (false coloured red) varied and was heterogeneous across the tissue surface. Surface coverage (median = bars, error = range) is summarised in **e**/**j** (*n* = 5). Increasing dose (rows) typically resulted in greater / deeper surface coverage, with only the far peripheries of the tissue remaining unexposed to nanoparticles at 1000 μg (**d**, **i**). Alternative dose metrics including the 3D equivalent total mass doses with area unit components are provided in Table 2

To characterise 3D topical exposures, particle deposition state was preserved immediately after dosing by vitrification in liquid nitrogen [43], and was subsequently imaged using cryogenic scanning electron microscopy (cryo-SEM) (Fig. 4). For both the 16 nm-SiO_2_ and 85 nm-SiO_2_, the lower total mass doses (≤300 μg) resulted in patchy, heterogenous surface coverage, with some areas of the tissue surface found to remain completely free of nanoparticles. With increasing dose (≥450 μg) however, coverage became increasingly layered and thick enough to mask the surface features of the underlying tissue. Areas of unexposed stratum corneum became less common and were found only at the far peripheries of the tissue surface for the highest (1000 μg) exposures. Further cryo-SEM images are presented in Additional files 5 and 6.

**Fig. 5:**
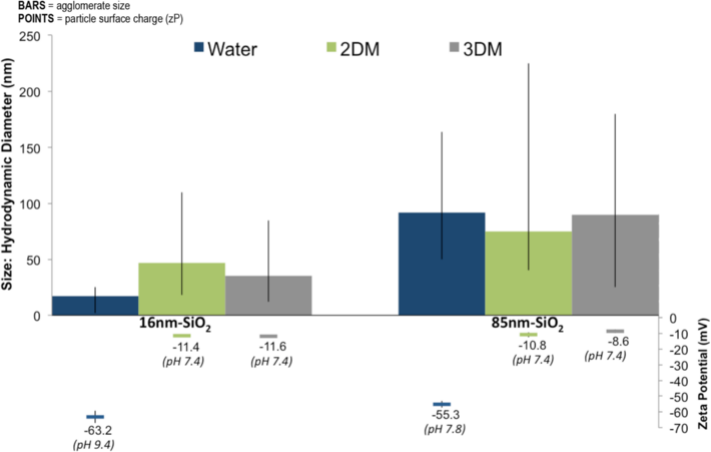
Characterising in-medium nanoparticle exposures using DLS: BARS = modal agglomerate hydrodynamic diameter (±99 % distribution range); POINTS = zeta potential (± SD). Size/charge was calculated by peak analysis of averaged number distributions (10 scans; *n* = 2). DLS was carried out at 37 °C for the 300 μg/mL (2D) and equivalent 3D (450 μg) doses. A growth medium reference without nanoparticles was also assessed during each replicate to ensure nanoparticles were being reliably detected against the serum particulate background. Alternative dose metrics including the 3D equivalent total mass doses with volume (in-medium exposures) unit components are provided in Table 2

The use of different growth media with the 2D and 3D models was unavoidable due to their different physiological requirements, and the need for specific growth factors to maintain the structure, growth and differentiation of the 3D model. In order to characterise the surface charge (zeta potential) and agglomeration state of the test articles examined using the 2D/3D assays with the in-medium exposures, DLS was carried out for the 2D (300 μg/mL) and equivalent 3D (450 μg) doses (Fig. 5). Often nanoparticle agglomerates form in cell culture growth media, complicating DLS interpretation as large particulates bias size distributions even when lowly abundant, since light scatter is proportional to the sixth-power of particle diameter [44–47]. An approach using the peak maxima (i.e., size mode) and size range spanned by 99 % of the frequency distribution by particle *number* was therefore used to compare agglomerate size distributions, and permit better correction for these factors [46].

Relative to water (manufacturer dispersant), in which agglomerates ranged in size from 9 – 32 nm (Table 1), the 16 nm-SiO_2_ showed evidence of increased agglomeration in both the 2D and 3D growth medium (M) types. Size ranges increased, spanning 18–110 nm in the 2DM and 12–85 nm in 3DM. The 85 nm-SiO_2_behaved similarly, with size ranges increasing in the 2DM to 40–225 nm and to 30–180 nm in the 3DM, when compared to dispersion in water (50–164 nm). Although the size ranges established were therefore consistently smaller in the 3DM compared to in the 2DM, this size difference was equivalent to the addition of approximately one primary particle to the measured agglomerate diameter. In all instances, particle incubation in either 2DM or 3DM resulted in the establishment of highly similar zeta potentials (around −10 mV).

### Comparing 2D and 3D assay dose-responses to the silica nanoparticles

**Fig. 6:**
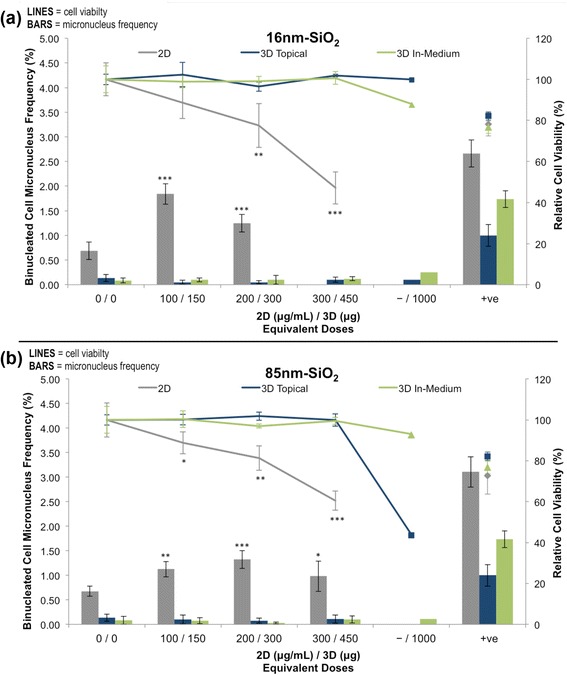
(Geno)toxicity assessment of silica nanoparticles exposed at equivalent doses to the 2D and 3D test systems: (**a**) 16 nm-SiO_2_, and (**b**) 85 nm-SiO_2_. BARS = micronucleus frequency; LINES/POINTS = cell viability. 2D cell cultures (2D) (*n* = 6, error bars = SD)/3D tissues (*n* = 2, error bars = range; except 1000 μg where *n* = 1) were exposed for 24 h in absence of cyt B via the 3D topical / in-medium or 2D exposure routes. Genotoxicity was assessed until cell viability decreased below 50 %. Equivalent 2D/3D doses were established by total mass dose normalisation according to the total number of cells in each culture model at time of inoculation (see Methods). (*) (**) (***) indicate statistical significance relative to control at *p* < 0.05, *p* < 0.01 and *p* < 0.001 respectively. Alternative dose metrics including the 3D equivalent total mass doses with area (topical exposures) and volume (in-medium exposures) unit components are provided in Table 2

2D and 3D responses to the 16 nm-SiO_2_ and 85 nm-SiO_2_ in terms of relative cell viability and binucleated cell MN frequency are presented in Fig. 6. Significant decreases in cell viability were found in the 2D assays at doses ≥200 μg/mL for the 16 nm-SiO_2_ (*p* < 0.0016) and ≥100 μg/mL for the 85 nm-SiO_2_ (*p* < 0.015). Furthermore, significant MN induction was found for all exposures of both particles (*p* < 0.002). Equivalent 3D exposures had no significant effect on 3D model viability or MN frequency regardless of exposure route (up to 450 μg) (*p* **>** 0.38). For this reason, a single 3D replicate dosed at 1000 μg was also examined. At this extreme, well above the 50 % cytotoxicity threshold for both particle types at equivalent dose in the 2D assay, no (geno)toxic response was observed for the 3D topical 16 nm-SiO_2_ and 3D in-medium 85 nm-SiO_2_ exposures. At this dose however, a small decrease in cell viability (88 % of control) and accompanying rise in MN frequency (2.8 fold) was detected for the 16 nm-SiO_2_ in-medium, and a sharp decline in cell viability (44 %) was noted for the 85 nm-SiO_2_ topical exposure. Due to the single replicate nature of these results, statistical analysis was not attempted and they are instead presented as preliminary findings to promote discussions regarding the importance of cellular uptake assessment in the avoidance of false positive results in 3D assays. The dose-response data used in the creation of Fig. 6 are provided in Additional file 7.

### Cell uptake: silica nanoparticle localisation in the intact 2D and 3D test systems

**Fig. 7:**
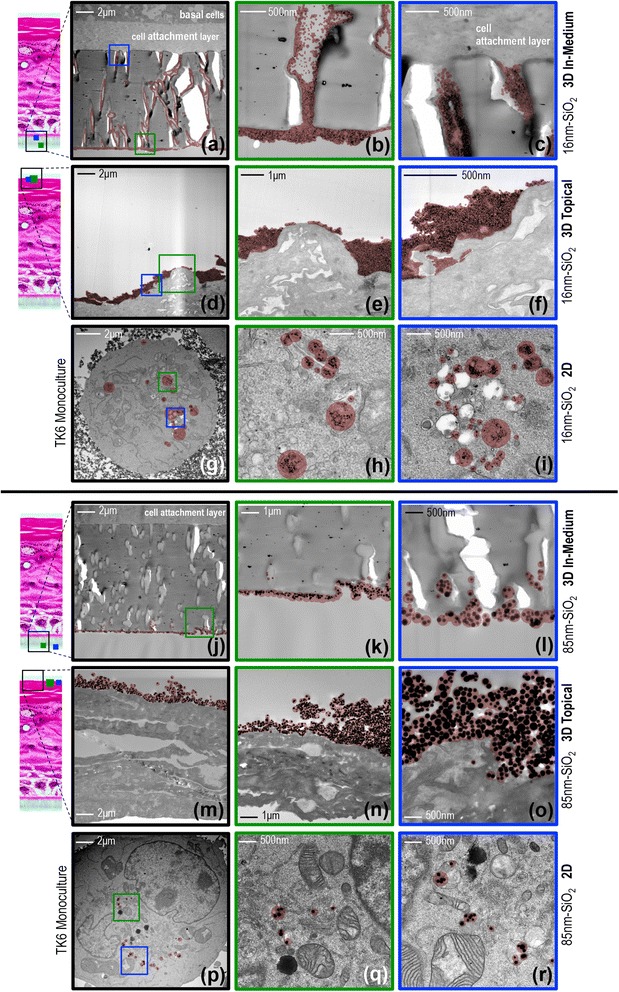
Contrast-inverted HAADF-STEM cross-sectional electron micrographs showing nanoparticle uptake in the 2D and 3D assays: Images were taken at the harvest time-point for the 300 μg/mL (2D) and equivalent 3D (450 μg) exposures. 16 nm-SiO_2_ (**a** – **i**); 85 nm-SiO_2_ (**j** – **r**). The inset light micrographs (left) show the position of the electron micrographs/particles (indicated, red outlines) in context of the complete tissue cross-section after dosing cell cultures (2D) or 3D tissue models via the in-medium or topical exposure routes (presented in triplicate by row). Magnified regions (green / blue outlines) are indicated on the low magnification images (left column, black outlines) where applicable. Alternative dose metrics including the 3D equivalent total mass doses with area (topical exposures) and volume (in-medium exposures) unit components are provided in Table 2

To investigate whether the response differences between the 2D and 3D assays related to differences in cellular uptake, nanoparticle localisation was investigated at the harvest time-point using inverted contrast high angle annular dark-field scanning transmission electron microscopy (HAADF-STEM) and EDX spectrometry (Fig. 7). This imaging mode was chosen as it is sensitive to atomic number, thus aiding nanoparticle detection, and because it provided excellent subcellular contrast without need for a secondary post-fixative (e.g., uranyl acetate) that, in our experience, can obscure nanoparticles [48]. Analysis was carried out for the 2D (300 μg/mL) and equivalent 3D (450 μg) exposures, the highest equivalent doses tested across both assays.

Analysis of the 16 nm-SiO_2_ in-medium exposures (Fig. 7a to c) showed extensive distribution of nanoparticles throughout the pores of the trans-well insert’s nylon membrane (schematically explained in Fig. 2b). However, no evidence of exposure/uptake to the basal cells immediately above the membrane was found, despite extensive imaging. For the topical exposure route (Fig. 7d–f), the 16 nm-SiO_2_ appeared largely unchanged from time of topical deposition (cryo-SEM, Fig. 4) with no evidence of penetration beyond the outmost layers of the stratum corneum. In contrast, 16 nm-SiO_2_ 2D exposures showed large numbers of particles in contact with cell membranes and internalised within vesicles (Fig. 7g–i). Similar results were found for the 85 nm-SiO_2_, except particles were only located along the bottom of the trans-well membrane, and not throughout it, after 3D in-medium exposure (Fig. 7j–l). Again, no evidence of subcutaneous particle penetration was found via the 3D topical route (Fig. 7m–o). Fewer cell-membrane bound particles were found in the 2D exposures (Fig. 7p–r); however, uptake still occurred with the 85 nm-SiO_2_ located in vesicles in the majority of cell sections imaged.

In overview, the particle uptake study showed no exposure to the dividing cells of the 3D models (i.e., for particle mass doses of ≤ 450 μg), whilst 2D exposures at equivalent mass per cell doses resulted in direct nanoparticle-cell contact and uptake. Close scrutiny of the 3D in-medium exposures suggests that the cell attachment layer (Fig. 7a, c) immediately between the insert membrane and basal cells (schematically explained in Fig. 2b) may have acted as a physical barrier preventing basal cell exposures. HAADF-STEM shows this layer is dense and lacks the pored structure of the trans-well membrane (Fig. 7a and c). No 2D-cell micrographs supported the possibility that nanoparticles were free in the cytoplasm or that direct nanoparticle-nucleus colocalisations occurred. Further images and EDX spectra for both assays are presented in Additional files 8, 9, 10, 11 and 12.

## Discussion

Commercially available 3D models offer the opportunity to assess the pathophysiologic activity of test articles in readily available, standardised systems that are more reflective of in vivo conditions. The EpiDerm™ model is available by overnight refrigerated shipment across North America and Europe, providing testing laboratories access to the same starting materials with proven inter-laboratory reproducibility [32]. In addition to genotoxicity assessment, protocols that permit use of EpiDerm™ tissues for skin irritation and corrosion assessment, as well as the study of dermal drug delivery and phototoxicity, have been developed indicating the broad potential of these models for the assessment of diverse toxicological endpoints [49–52].

Our experience has shown that one trained user can expose and harvest up to 48 tissues within the four-day nano-RSMN period, thus 3D in vitro assays can offer significantly higher throughput than equivalent in vivo tests. Although the manual scoring of resultant slides remains a laborious process, we have previously reported our successful use of the MetaSystems automated slide scanning platform to increase RSMN scoring throughput via semi-automation [36], and efforts to permit fully automated scoring by flow cytometry are underway [53].

Although there have been recent efforts to validate the 3D RSMN assay for chemicals, as with many test systems, simply applying these chemical-specific methods to nanomaterials was not possible [11, 28, 29, 31, 32]. Thus, this work evaluated the utility of MatTek Corporation’s EpiDerm™ 3D human epidermis model for genetic toxicity assessment of engineered nanomaterials. Alongside the general requirement of appropriate dosimetry to permit 2D/3D model comparison, key issues identified in applying the chemical-RSMN method to nanomaterials included: (1) requirement of a sequential cyt B regime, (2) a dosing regime that does not disrupt the ALI, (3) requirement for methods to characterise in-medium and ALI exposures, and (4) requirement for methods to understand penetration and/or uptake throughout the 3D model.

As already noted, TK6 cells were chosen for the 2D assays conducted in this study on the basis that they are recommended in the OECD test guideline for the in vitro micronucleus assay (i.e., Test Guideline 487) [42]. Their utility for internationally-accepted, regulatory evaluations is based upon: their human origin (i.e., as opposed to rodent), the availability of sufficient historical control data to permit results validation (i.e., both intra- and inter-laboratory), their proven ability to sustain log-phase growth throughout the assay period, their known maintenance of a stable karyotype, and their well characterised DNA repair capacities and P53 competency [42]. Collectively, these characteristics have been shown to reduce the incidence of ‘misleading positives’ relative to what has been observed for other cell lines [21, 54, 55]. Therefore, though dermal cells could have been used for the current study, their use for in vitro micronucleus frequency assessment would constitute a significant deviation from the aforementioned, internationally-validated testing strategy that is routinely employed for regulatory evaluations and subsequent decision-making. As such, use of TK6 cells was considered the best starting point to permit comparison of a standard, validated in vitro assay approach with a new version employing 3D in vitro models and suitably adapted test methods for nanomaterial test articles.

To date, most RSMN studies have reported dose (i.e., topically-applied) in mass per volume units [22, 32, 33, 56]. In the context of the chemical-RSMN method [30], this means 10 μL of a chemical test article at e.g., 50 μg/mL is applied twice, at 24 h intervals. In total, the tissue therefore receives 1 μg total mass of the test article, but the exposure metric is reported as 50 μg/mL. In contrast, a 2D assay using typical 10 mL suspension monocultures dosed at 0.1 μg/mL would also utilise a total mass dose of 1 μg. Thus, the current chemical-RSMN approach to reporting dosimetry complicates 2D to 3D comparison for three key reasons: (1) multiple exposures are not represented, (2) after vehicle evaporation, a topically applied dose per unit volume is no longer meaningful, and (3) differences in the quantity and three-dimensional arrangement of the cells receiving the dose dramatically influences the probability of test article contact. In an attempt to make the cellular architectures of the different test systems under comparison the key differentiating factor, and to provide more comparable 2D and 3D dosimetry, this study normalised the total mass of nanoparticles administered to the number of cells in the respective assay format. This approach was previously applied in our laboratory to examine the relative sensitivities of 2D monocultures and the RSMN assay to a range of chemical test articles [36].

In reality of course, any approach to this 2D/3D dose normalisation will have limitations in one respect or another as the exposures are *fundamentally different* between the two systems: the 3D topical exposures are entirely deposited onto the in vitro model after vehicle solvent evaporation, whilst in-medium deliveries require diffusion/sedimentation processes for cellular contact. As no perfect, unbiased way to address these differences in exposure likely exists, the importance of a comprehensive set of tools to understand exposure and cellular uptake, as demonstrated herein, becomes increasingly apparent. Ultimately, they provide the means to interpret differences in 2D/3D dose-response results in the context of the limitations of any employed dose-normalisation approach.

Cyt B inhibits actin polymerisation, which is required for endosome formation in the active cellular uptake pathways known to be important for nanoparticle internalisation. Sequential cyt B addition (i.e., nanomaterial exposure in absence of cyt B prior to sequential addition) is therefore important for cytokinesis blocked MN assessments using nano test articles [11, 28, 29]. Importantly, the accommodations made for this herein (i.e., dosing for 24 h without cyt B prior to its addition for 42 h) had no effect on tissue viability or binucleate frequency. This finding is supported by other chemical-RSMN studies that have made similar adaptations to increase exposure time to augment metabolism of chemical test articles [32]. Tissue growth, differentiation, structure and binucleate frequencies determined here for the nano-RSMN method are also highly similar to those reported for the original chemical-RSMN approach [22, 32, 33, 56].

Topical exposure to water is known to negatively impact EpiDerm™ viability with this finding attributed to ALI disruption [36]. For this reason, 10 μL acetone, a commonly used dosing vehicle in dermal rodent studies, is the recommended chemical-RSMN dosing vehicle [33]. Here it was also possible to administer the silica nanoparticle test articles in acetone, without attendant disruption of colloidal stability [57]. An optimal solvent vehicle for the nano-RSMN assay should therefore: (1) evaporate to maintain the ALI, (2) promote colloidal stability and not degrade the test nanomaterial, (3) not cause decreased viability or induce genotoxicity, and (4) yield acceptable tissue surface coverage upon deposition. Although acetone was a suitable vehicle for this study, it will not be a universal vehicle since colloidal dispersion stability and the degradation potential of nanomaterials depends on their specific composition and physico-chemical properties [58–60]. Investigation of the range of solvents compatible with the chemical-RSMN assay has already been suggested in order to broaden its utility and application domain [33]. To date, multiple vehicles including diluted ethanol, dimethylsulphoxide, acetone:olive oil, water, saline and acetone have been tested for compatibility with some successes [33, 36, 56]. Other options, which may be suitable for nanomaterials include aerosol delivery methods, analogous to those employed for inhalation exposures for (nano)particle toxicity assessment [61–64]. Commercial systems designed for in vitro ALI exposures are also available, indeed, they have been already been successfully used for gases and complex, particle-containing aerosols [65, 66].

It is now well appreciated that comprehensive physico-chemical characterisation is important for the interpretation of any nanosafety study. Developing methods to assess nanomaterial exposures for both in-medium and topical ALI dosing regimes was therefore essential for the development and establishment of nano-RSMN, and facilitated comparisons between the 2D and 3D assay results presented here.

For both 2D and 3D in-medium exposures, DLS analysis showed nanoparticle zeta potentials universally increased to similar charges close to isoelectric zero where colloidal dispersions are typically most prone to agglomerate [67, 68]. Accordingly, particle size ranges increased, suggesting decreased electrostatic repulsion between individual particles due to negative surface charge shielding by serum protein interaction elevated agglomeration [15, 69, 70]. Despite this evidence for the formation of some agglomerates, the particle size range present in 3DM showed that the skin models were still exposed to a size fraction of individual nanoparticles via the in-medium dosing route. This provides further evidence that the cell attachment layer constituted a barrier and thus a confounding error to 3D in-medium exposures, as even single particles ≤16 nm were unable to pass through it and access the apical cell layers.

Exposure characterisation after dosing topically was also critical for three key reasons: (1) it quantified that the deposition method resulted in acceptable surface coverage with the test nanomaterial; (2) it has the potential to indicate unwanted agglomeration or particle degradation due to choice of an unsuitable dosing vehicle; (3) it indicated the dose above which the ALI became submerged by the nano test article. With regard to the final point, as no evidence of 3D topical penetration was found, ALI disruption is considered the most likely explanation of the cell viability decreases observed in response to the 1000 μg exposures; given the near 100 %, layered surface coverage evidenced during exposure characterisation by cryo-SEM.

Scrutiny of nanoparticle penetration and cellular uptake was found vital to dose-response interpretation, with the uptake studying showing that: (1) 3D topical exposures did not penetrate the stratum corneum skin-barrier, (2) 3D in-medium exposures were likely prevented from reaching the apical cells due to the impermeability of the collagen-coated cell attachment layer, and (3) 2D in-medium exposures contacted cells and were taken up in vesicles. Dose-dependent (geno)toxicity was therefore associated with increasing 2D cellular exposures, and was absent in 3D models (≤455 μg) since live-cell nanomaterial exposure did not occur in 3D. In the absence of corresponding uptake results it was also not possible to explain the 3D responses observed for 1000 μg exposures. It seems unlikely that increasing dose suddenly facilitated skin-barrier penetration and living cell exposures; rather, it seems more likely that ALI suffocation (i.e., inhibition of gas exchange) from excessive particle coverage, or failure to restart active tissue proliferation (as previously observed [36]) are more likely explanations. Though limited information regarding real-world dermal exposure concentrations to nanomaterials exists, it seems reasonable to assert that these 1000 μg exposures were likely extreme. Nevertheless, the OECD test guideline for the in vitro micronucleus assay states that dose should be increased with the aim to reach 55 ± 5 % cytotoxicity at the highest tested concentration [42]. Care to not mistake ALI suffocation for test article toxicity when following this guideline recommendation may therefore prove important with nano-RSMN, and full determination of the causes of toxicological responses at very high apical exposures seems to be a productive area for subsequent investigation.

In light of the uptake study results, and their implications regarding the utility of the RSMN for nanosafety assessment, it is essential to discuss the ability of 3D reconstructed skin models to represent real human skin. Although current research is limited, a study of quantum dot nanoparticles (6 nm; ≤ 24 h exposure) showed similar, limited penetration in both human skin in vivo and EpiDerm™ when compared side-by-side [40]. However, other dermal studies in vivo show that nanomaterials can penetrate the epidermis, enter circulation and induce organ toxicity, but the process typically requires > 30 days continuous exposure [71], or compromised skin-barrier integrity [72]. Micron-scaled particles are also reported to penetrate the stratum corneum more readily at the site of hair follicles [5, 73]. Although EpiDerm™ does not contain these follicular structures, and exposures are limited to the 72 h during which its 3D morphology can be retained, these limitations are arguably typical caveats associated with any in vitro test system. Interestingly, wound healing has been studied using reconstructed skin models, suggesting that the impact of compromised barrier integrity on nanoparticle (geno)toxicity could be studied in vitro [74, 75]. Herein, the negative 3D (geno)toxicity results after topical exposures were found related to the 3D model’s skin-barrier properties, which restricted interactions between the dividing cells and the test article. Whereas further work to establish how well the barrier properties of EpiDerm™ reflect in vivo skin is required, it seems reasonable to assert that these results are more reflective of actual human hazard than traditional assays using 2D monocultured cells.

Although investigating the (geno)toxic potential of amorphous silica was a secondary aim to developing nano-RSMN, a brief commentary on the positive 2D (geno)toxicity findings is appropriate. Although comparisons of nano(geno)toxicology studies is complicated by the different exposure durations, dose-ranges, cyt B regimes and cytotoxicity methods used, a review of twenty-one in vitro studies using amorphous silica found nineteen that reported decreases in cell viability similar to those found here [41]. Of specific interest, identical Levasil® particles caused similar ≤ 22 % and ≤ 36 % decreases (16 nm and 85 nm-SiO_2_, respectively) in primary human lymphocytes (24 h exposure, 31.6 – 316 μg/mL) [76]. Therefore, the 2D cell viability decreases found here are well-aligned with the majority of reported findings.

Fewer silica studies have thus far considered the MN endpoint in vitro. An earlier study showed that 34 nm silica nanoparticles caused ≤ 1.7-fold increases in MN frequency at ≤ 400 μg/mL (mouse fibroblasts; 24 h cyt B co-exposure), and similarly, 16 nm, 60 nm and 104 nm silica particles caused fold increases of ≤ 1.8 at ≤ 300 μg/mL in human lung carcinoma cells (sequential regime, 4 h +36 h cyt B) [77]. Other in vitro MN studies have not shown increases in MN frequency however [76, 78]; interestingly, identical Levasil® nanoparticles did not cause significant MN induction (≤1000 μg/mL) in primary human lymphocytes (24 h co-exposure and sequential 4 h +20 h cyt B methodologies utilised ) [76]. This study’s findings of 2D MN induction in the order of ≤ 3.0-fold are therefore higher than those previously reported. This likely relates to both the cell line selection and cyt B regime used. The TK6 cell line used here is validated for the MN assay, whilst other investigations used rodent cells or oncogenically transformed cells that have differing sensitivities and varying abilities to cope with genotoxic stress [21, 79, 80]. The exposure period for uninhibited nanoparticle uptake (without cyt B) was also substantially longer (24 h) in this study in comparison with other investigations (≤4 h). Whereas further research is required to delineate the mechanism(s) underlying the 2D chromosomal damage observed herein, previous studies in other mammalian cell types indicate vesicularly-internalised nanoparticles can generate reactive oxygen species, initiate oxidative stress or mechanically hinder mitotic processes [81–85]. It seems reasonable to assert that similar mechanisms are operating here.

Although the MN assay is the ‘gold standard’ test for the detection of most forms of chromosomal damage that will be transmitted to the next generation of cells (i.e. non repairable damage), it is not sensitive to some forms of ‘low-level’ DNA damage including single-strand breaks, alkali labile sites, DNA-DNA and DNA-protein cross-linking that might be induced by nanoparticle-mediated oxidative damage mechanisms [86]. In this regard, it is worth noting validation of the EpiDerm™ 3D model for the single cell gel/comet assay, which is sensitive to these forms of damage, is underway for chemical test articles. At this stage however, considerable intra- and inter-laboratory variation is currently reported [53], suggesting further optimisation of the 3D assay method will be required before similar adaptation for nanomaterials becomes an avenue for future research.

## Conclusions

This study successfully developed and employed a 3D ‘nano-RSMN’ method using nano test articles to which dermal exposure is a concern. Although complex and challenging, rigorous exposure characterisation and uptake assessment are critical for successful interpretation of in vitro (geno)toxicity results. Thus, to complement genotoxicity assessment, a package of tools (i.e., DLS and cryo-SEM, HAADF-STEM and EDX) with wide-ranging applicability to other 2D/3D model comparisons were employed and demonstrated effective for the examination of nanomaterial behavior in the 3D in vitro test environment.

These characterisations revealed the specific importance of the 3D model’s skin-barrier layer in modulating living-cell exposures to dermally applied nanomaterials, with the 3D RSMN arguably better reflecting real-world exposures when compared to traditional 2D monoculture approaches. Although it seems reasonable to assert that this 3D in vitro system might provide more appropriate nanomaterial safety assessments, it is not possible at the present time to discount the involvement of wound- or follicle-facilitated exposures of dividing dermal cells in adverse effects. Further clear and definitive comparisons of nanomaterial penetrations in vivo and EpiDerm^TM^ will therefore be required to fully evaluate nano-RSMN performance. It is also apparent that further development of exposure methods that are uniformly compatible with a wide range of nanomaterials, yet able to deliver exposures without air-liquid interface compromise, will be essential to permit full exploration of the utility of 3D models for nanotoxicology testing.

In future nanosafety testing strategies, it is suggested that 2D and 3D in vitro assays might prove complimentary. The potential for DNA damage upon exposure to the parenchymal cell type of interest could be indicated using 2D exposures, then 3D models of exposure route(s) of interest could be used to ascertain how likely such exposures are to occur. In the short term however, a more realistic objective seems to be the development of 3D in vitro models to the point where they permit better prioritisation of animal testing according to exposure risk; facilitating animal reduction through informed study design.

## Methods

### General preparation of chemicals and nanomaterials

Chemicals were purchased from Sigma-Aldrich (Gillingham, UK) unless otherwise stated. The amorphous silica nanoparticles Levasil® 50 (85 nm-SiO_2_) and Levasil® 200 (16 nm-SiO_2_) were donated by Dr. R. Landsiedel (BASF, Germany). Filter-purified water was prepared by the Milli-Q purification system (Merck Millipore, Nottingham, UK).

### Cell culture

TK6 human B lymphoblastoid cells (Health Protection Agency, Wiltshire UK) were cultured in RPMI 1640 containing 1 % L-glutamine (GIBCO, Paisley UK) and 10 % horse serum (2DM) (BioSer, Sussex UK). EpiDerm™ tissues (#EPI-200-MNA, MatTek, Bratislava, Slovakia) were delivered by overnight refrigerated shipment and maintained using New Maintenance Growth Media (3DM) (#EPI-100-NMM, MatTek). Cell cultures were incubated at 37 °C in humidified, 5 % CO_2._

### Preparation of nanomaterials for biological experiments

Stock nanoparticle solutions were briefly vortexed then mixed 1:1 with medium (2D assays) or reagent grade acetone (3D assays). Solutions were then dispersed (approx. 288 J/mL) using an ultrasonic bath (#FB15048, Thermo-Fisher, Loughborough, UK). Dosing was carried out immediately from these solutions which were prepared fresh for each experimental replicate.

### Equivalent dosing: 2D to 3D assay extrapolation

The mass/volume 2D dosing approach was not suitable for the 3D models as topical exposures required evaporation deposition to maintain the ALI. 2D/3D dose was therefore made equivalent in terms of total nanoparticle mass to total cell number. E.g., for a 2D dose of 100 μg/mL, each 10 cm^3^ culture (#690175 T25 flask, Greiner Bio-One, Monroe USA) had ~2x10^6^ cells at time of dosing and received a total nanoparticle mass of 1000 μg. By contrast, EpiDerm^TM^ tissues (surface area 0.64 cm^2^) contained just ~3x10^5^ cells (determined at harvest after trypsinisation into an individual cell suspension by haemocytometer count; mean = 304,000; SD = 34,000; *n* = 8). Therefore to obtain an ‘equivalent’ mass dose per cell in 3D; a total nanoparticle mass dose of (1000/6.6) = ~150 μg was required (as there were 2x10^6^/3 × 10^5^ = 6.6 fewer cells). Alternative dose metrics including the 3D equivalent total mass doses with area (topical exposures) and volume (in-medium exposures) unit components are provided in Table 2.

### Physico-chemical characterisation: DLS and TEM preparation

Characterisation was carried out at 300 μg/mL (2D) or equivalent 450 μg (3D) concentrations. TEM samples were prepared for primary characterisation by drop-casting aqueous solutions onto glow-discharge treated, standard holey carbon TEM support grids (Agar Scientific, Stansted, UK). Nanoparticle primary size distributions were constructed from individual particle diameter measurements (n >120) made using ImageJ 1.48v [87]. Agglomerate size (hydrodynamic diameter) and surface charge (zeta potential) were measured by DLS using a ZetaSizer Nano ZS instrument (Malvern Instruments Ltd, Worcestershire, UK). 2D and 3D culture medium dynamic viscosities (at 37 °C) were determined as 0.757 cP and 0.722 cP respectively by U-tube viscometer method, and both had a measured refractive index of ~1.34. To gain accurate size information, care was taken to reproduce the process used to expose cells. 500 μL of each dispersion was then loaded into a capillary cell (#DTS1061, Malvern). Samples were allowed to equilibrate to 37 °C for 2 min prior to measurement. Size measurements were calculated from the average of ten 100 s scans, and charge measurements from the average of ten 10 s scans, with measurements repeated in duplicate from independently prepared dispersions. Particle size was represented by the peak (modal size)/size range spanned by 99 % of the frequency distribution by number. A blank growth medium reference was included in all analyses to ensure particles were reliably detected. The dispersant dielectric constant was assumed to be 74.5, and Henry’s function was set at the Smoluchowski approximation of F(ka) = 1.5.

### Transmission electron microscopy

TEM was conducted at 200 kV using a Tecnai F20 FEG-TEM fitted with a high angle annular dark field (HAADF) detector (FEI, Eindhoven, The Netherlands) and an INCA 350 EDX system with a 80 mm^2^ silicon drift detector (Oxford Instruments, Abingdon UK). Images were recorded using an Orius SC600A CCD camera (Gatan Inc., Abingdon, UK).

### 2D RPD and MN assays

The effect of silica nanoparticle exposure to the TK6 cells in terms of cell growth / cytotoxicity was assessed by relative population doubling (RPD) analysis, with genotoxicity assessment conducted alongside in duplicated satellite flasks by cytokinesis blocked MN assay (*n* **=** 6; separated across days). Each replicate started by thawing a new vial of passage seven cells which were cultured for at least 96 h prior to initiation of experiments. On day one, 10 mL cell cultures were seeded in T25 flasks (#690175 Greiner Bio-One) at 2x10^5^ cells/mL (count 1) and returned to the incubator for 4 h prior to dosing. Cells were then exposed for 24 h before washing twice with phosphate-buffered saline (PBS) (GIBCO) and left to recover for one cell cycle (17 h) (count 2) in the presence of 3 μg/mL cyt B (#C6762, Sigma) (MN assay). RPD (%) was calculated as (no. population doublings (PD) in treated cultures / no. PDs in control cultures) x 100; where PD was defined as (log10(count2/count1))/log(2). Mitomycin C (MMC) (0.01 μg/mL) (#M7949 Sigma) was used as a positive control. Cells were harvested for MN analysis by resuspension in 0.56 % potassium chloride hypotonic solution followed immediately by 10 min incubation in ‘fixative one’ (5:1:6 methanol-acetic acid-0.9 % sodium chloride) at 4 °C. Cells were then washed through four 10 min changes of fixative two (5:1 methanol-acetic acid; 4 °C), with the cells left in the final wash overnight at 4 °C. 100 μL volumes of the fix/cell suspension were then pipetted onto polished, hydrated slides. Slides were allowed to air dry, then counterstained with 0.15 μg/mL 4’,6-diamido-2-phenylindole (DAPI) nuclear stain. The presence of micronuclei in binucleated cells (six replicates, 2000 binucleates each) was then assessed automatically using a Axioimager Z2 fluorescent microscope, 1MP CCD camera (Carl Zeiss, Cambridge, UK) and Metafer 4 software (MetaSystems Group, Altlussheim, Germany), using image classifiers previously optimised for TK6 [23]. All micronucleated cells were manually validated using the 100X objective.

### 3D RSMN assay

Upon arrival (+0 h), 900 μL 3DM was aliquoted into each well of four six-well plates. Moisture was removed from tissue surfaces using sterile gauze before adding the tissues to the plates and returning to the incubator for four hours. Levasil® and mitomycin C (MMC) positive controls (total mass 0.06 μg topical or 0.03 μg in-medium) were administered in 10 μL acetone (*n* **=** 2) using a positive displacement pipette (Gilson Scientific, Luton, UK). Plates were immediately tilted to distribute the dose across the tissue surface (0.64 cm^2^/9 mm diameter). After 24 h incubation, 3DM was aspirated and re-fed containing 3 μg/mL cyt B with this step repeated for a second time 24 h later. Tissues were then returned to the incubator for 18 h prior to harvest (+66 h) using the protocol described in Curran et al.*,* [30]. Trypsinised cells were then fixed using ice cold 3:1 methanol:acetic acid (Thermo-Fisher Scientific). 10 μL volumes of this fix/cell suspension were then pipetted onto polished, hydrated slides. Slides were stained by immersion in acridine orange (#A8097, Sigma) diluted to 40 μg/ml in Gurr buffer at pH 6.8 (#10582-013, GIBCO), then MN frequencies were assessed manually under UV light using a BX50 fluorescent microscope (Olympus, Southend-On-Sea, UK). Two replicates of 2000 binucleated cells were scored for micronuclei with cell viability assessed according to binucleated/mononucleated cell frequencies.

### Haematoxylin and eosin tissue staining

Tissues were fixed by immersion in 4 % paraformaldehyde in PBS for 15 min at 37 °C before changing to fresh fixative at 4 °C for 15 min. Tissues were then excised from trans-well inserts using a scalpel, sandwiched between sheets of filter paper and placed in a Shandon Tissue Cassette (#B1003500AQ, Thermo-Fisher Scientific). Cassettes were stored in PBS before automatic overnight processing to paraffin wax at the Cellular Pathology Department of Singleton Hospital (Swansea, UK). Cycles first involved dehydration by ethanol series before three changes of 100 % xylene (at 30 °C). Tissues then underwent three changes of paraffin wax (at 62 °C). Processed blocks were sectioned at 3 μm thickness using a rotary microtome (#RM2235, Leica Microsystems, Milton Keynes, UK) and laid onto polished slides. To stain, slides were first dewaxed and rehydrated by baking at 60 °C for 1 h before changing twice through xylene, a reverse ethanol series and 1 min in water. Slides were then placed in 0.01 % haemotoxylin (#LAMB/170-D, Thermo-Fisher Scientific) for 3 min. A drop of 0.01 % eosin (#LAMB/100-D, Thermo-Fisher Scientific) was then added for 5 s before immediately washing in water. Slides were then dehydrated through an ethanol series then oven dried for 15 min at 60 °C. Finally, slides were immersed in xylene before a drop of DPX mounting medium (#44581, Sigma) was added to secure coverslips. Tissues were imaged using a BX51 light microscope equipped with a 3.2 MP Colour View 1 CCD camera and CellSens Entry software (Olympus, UK).

### Cryogenic scanning electron microscopy

The preparation chamber and stage of the S-4800 scanning electron microscope (Hitachi GmbH, Maidenhead, UK) were purged with liquid nitrogen (3 L/min) to facilitate cooling to between -150 and -160 °C. Gas control was managed using a Polaron Range #PP7483 gas control unit, #PP7482 turbo pump control unit and #PP7480 Cryo-SEM preparation control unit (Quorum Technologies, East Sussex, UK). Immediately after dosing, tissues were mounted by their insert membranes onto SEM stubs (#AGG3026, Agar Scientific) using colloidal graphite adhesive (#G303, Agar Scientific). The mounted specimen stub was then plunged into nitrogen slush to facilitate vitrification before transfer via a vacuum capsule into the pre-cooled preparation chamber of the microscope. To enhance the contrast of the secondary electron images obtained at low accelerating voltages, the electron conductivity of the tissue surface was modulated by sputter coating with platinum. The preparation chamber was purged with argon at a pressure of 5x10^-2^ mbar such that a plasma discharge was observed, before coating with a sputter current of 5-10 mA for 30 s. To avoid damage and movement of the specimen under cryogenic conditions, imaging was subsequently conducted under a low 1.0 kV accelerating voltage in scanning mode. Surface coverage (after normalisation according to image area) was assessed by masking and image analysis using ImageJ 1.48v [87]. In the presented micrographs, Levasil® particles were false-coloured red to facilitate visualisation.

### Cell/tissue preparation for TEM

In the following steps, all buffers were 200 mM and maintained pH 7.3. TK6 cells were fixed in 2.5 % Millonig’s buffered glutaraldehyde (#R1314, Agar Scientific) for 15 min whilst EpiDerm™ tissues were fixed in Millonig’s buffered 2.5 % glutaraldehyde, 2 % paraformaldehyde for 30 min (37 °C). The respective fixatives were then replaced and cells/tissues incubated for a further 4 h at 4 °C. Excised tissues/pelleted cells (max RPM) were post-fixed in 1 % phosphate buffered osmium tetroxide (#R1016, Agar Scientific) for 2 h in the dark at 4 °C. Samples were dehydrated through an ethanol series before undergoing two changes of 100 % propylene oxide. Resin infiltration involved a 90 min incubation with a 1:1 ratio of medium resin (#T028, TAAB, Aldermaston, UK) to propylene oxide, overnight incubation in fresh 100 % resin on a rocking platform at 4 °C, and then baking for 16-24 h in fresh resin. Resin blocks were trimmed and ultrathin sections cut at 500 μm^2^ x 70 nm thickness using an EM-UC7 ultramicrotome (Leica Microsystems) and an Ultra 45° diamond knife (Diatome, Biel, Switzerland). Sections were picked up on 150 square mesh copper grids (#G2150C, Agar Scientific) and sputter coated with ~3.5 nm carbon (#Q150-TE, Quorum Technologies) prior to brightfield TEM/EDX spectroscopy and HAADF imaging in scanning transmission mode. In the presented micrographs HAADF images were contrasted inverted for easy comparison with bright field TEM images and Levasil® particles were highlighted red to facilitate visualisation.

### Statistical analyses

Dose-response significance was assessed using the framework laid out by Johnson et al.*,* [88]. Response data were assessed for homogeneity of variance and distribution normality by Bartlett and Shapiro-Wilk tests, respectively, after log_10_ transformation. If the transformed data passed these tests (*p* > 0.05), comparisons to negative controls employed one-sided (MN) or two-sided (cell viability) *post hoc* Dunnett’s test with alpha set at 0.05. Datasets that failed either test (*p* < 0.05) were analysed using the non-parametric *post hoc* Dunn’s test. Statistical analyses were conducted using the online tool DRSMOOTH via Swansea University’s Mutation Analysis Informatic Tools website (MutAIT.org) [89].

## Additional files

Additional file 1: Brightfield TEM micrographs of the BASF Levasil® silicon dioxide nanoparticles: 16 nm-SiO_2_ (a - d), 85 nm-SiO_2_ (e - h). (TIF 4314 kb)

Additional file 2: Primary Levasil® size distributions calculated from TEM micrographs: 16 nm-SiO_2_ (**a**), 85 nm-SiO_2_ (**b**). (TIF 276 kb)

Additional file 3: Levasil® EDX spectra relative to background: 16 nm-SiO_2_ (**a**), 85 nm-SiO_2_ (**b**). Regions analysed shown in the inset image. Copper and carbon signals originate from the TEM grid and its support film. (TIF 1453 kb)

Additional file 4: Binucleate frequency comparisons between different 3D RSMN methods, cyt B regimes and solvent (acetone) inoculation routes: The optimised nano RSMN protocol designed to permit topical or in-medium nanoparticle exposures yielded highly similar frequencies of binucleated cells to the original protocol designed for chemical test articles (*n* = 3, error bars = SD). (TIF 218 kb)

Additional file 5: Characterising 16 nm-SiO_2_ (false coloured red) topical 3D model deposition in acetone using cryogenic freezing and scanning electron microscopy: Dose increases down rows; images presented from separate samples prepared in triplicate. Alternative dose metrics including the 3D equivalent total mass doses with area (topical exposures) unit components are provided in Table 2. (TIF 7845 kb)

Additional file 6: Characterising 85 nm-SiO_2_ (false coloured red) topical 3D model deposition in acetone using cryogenic freezing and scanning electron microscopy: Dose increases down rows; images presented from separate samples prepared in triplicate. Alternative dose metrics including the 3D equivalent total mass doses with area (topical exposures) unit components are provided in Table 2. (TIF 9112 kb)

Additional file 7: Tabulated dose-response data used in the creation of Fig. 6: Human B lymphoblastoid cells (TK6) were exposed to the 16 nm-SiO_2_ or 85 nm-SiO_2_ for 24 h in absence of cyt B via the 3D topical (3D-T), 3D in-medium (3D-M) or 2D in-medium (2D) exposure routes. Genotoxicity (micronucleus frequency) was assessed until cell viability decreased below 50 %. Equivalent 2D / 3D doses were established by total mass dose normalisation according to the total number of cells in each system at time of inoculation (see Methods). Alternative dose metrics including the 3D equivalent total mass doses with area (topical exposures) unit components are provided in Table 2. (CSV 4 kb)

Additional file 8: Contrast inverted HAADF-STEM cross-sectional micrographs showing 16 nm-SiO_2_ nanoparticle localisation (indicated, red outline) in the 3D skin model: Images for each exposure route (450 μg exposures) are presented alongside undosed negative controls at the harvest time-point (+66 h) (images presented in triplicate by row). The inset light micrographs (left) show the position of the electron micrographs/particles in context of the complete tissue cross-section. Magnified regions (green/blue outlines) are indicated on the low magnification images (left column, black outlines) where applicable. Alternative dose metrics including the 3D equivalent total mass doses with area (topical exposures) and volume (in-medium exposures) unit components are provided in Table 2. (TIF 5694 kb)

Additional file 9: Contrast inverted HAADF-STEM cross-sectional micrographs showing 85 nm-SiO_2_ nanoparticle localisation (indicated, red outline) in the 3D skin model: Images for each exposure route (450 μg exposures) are presented alongside undosed negative controls at the harvest time-point (+66 h) (images presented in triplicate by row). The inset light micrographs (left) show the position of the electron micrographs/particles in context of the complete tissue cross-section. Magnified regions (green/blue outlines) are indicated on the low magnification images (left column, black outlines) where applicable. Alternative dose metrics including the 3D equivalent total mass doses with area (topical exposures) and volume (in-medium exposures) unit components are provided in Table 2. (TIF 5412 kb)

Additional file 10: Contrast inverted HAADF-STEM cross-sectional micrographs showing 16 nm-SiO_2_ nanoparticle uptake (indicated, red outline) in 2D TK6 lymphoblastoid cells: Images were taken at the harvest time-point (+42 h) (300 μg/mL dose). Efforts were made to image >50 cell sections. Particles could be seen surrounding / bound to cell membranes (a – c, black) and where readily internalised (red outlines) within vesicles (d – l) suggesting active uptake. Particle composition/uptake was confirmed by EDX spectroscopy. (TIF 7535 kb)

Additional file 11: Bright field TEM cross-sectional micrographs showing 85 nm-SiO_2_ nanoparticle uptake (indicated, red outline) in 2D TK6 lymphoblastoid cells: Images were taken at the harvest time-point (+42 h) (300 μg/mL dose). Efforts were made to image >50 cell sections. Particles could be seen surrounding / bound to cell membranes (a – f, black) and where readily internalised (red outlines) within vesicles (g – l) suggesting active uptake. Particle composition/uptake was confirmed by EDX spectroscopy. (TIF 8426 kb)

Additional file 12: Confirming 2D Levasil® uptake using EDX spectroscopy: (a) 16 nm-SiO_2_, and (b) 85 nm-SiO_2_. Areas analysed are shown in the inset images, with resulting spectra overlaid to compare background regions (black) to those containing nanoparticles (red). The copper signal originates from the TEM grid, and the carbon and osmium signals from the sample preparation method (e.g., the osmium tetroxide fixative used to enhance contrast and the resin used for embedding). (TIF 1653 kb)

## Abbreviations

16 nm-SiO_2_: BASF Levasil® 200 silicon dioxide nanoparticles
2DM: 89 % RPMI 1640, 10 % horse serum, 1 % glutamine used to culture TK6 cells in 2D assays
3DM: MatTek Corporation’s New Maintenance growth medium used to culture the 3D EpiDerm™ tissues
85 nm-SiO_2_: BASF Levasil® 50 silicon dioxide nanoparticles
ALI: Air-liquid interface
chemical-RSMN: Reconstructed skin micronucleus assay protocol developed previously [30] for chemical test articles
cryo-SEM: Cryogenic vitrification and scanning electron microscopy
cyt B: Cytochalasin B
DLS: Dynamic light scattering
EDX: Energy dispersive X-ray spectrometry
HAADF-STEM: High angle annular dark-field scanning transmission electron microscopy
MN: Micronucleus
nano-RSMN: Reconstructed skin micronucleus assay protocol developed here for nano test articles
RPD: Relative population doubling
RSMN: Reconstructed skin micronucleus
TEM: Transmission electron microscopy
