# Supplementary figures and images for: Genetic toxicity assessment of engineered nanoparticles using a 3D in vitro skin model (EpiDerm™)
Source: Part Fibre Toxicol. 2016 Sep 9;13:50. doi: 10.1186/s12989-016-0161-5 (PMC5016964; doi:10.1186/s12989-016-0161-5)

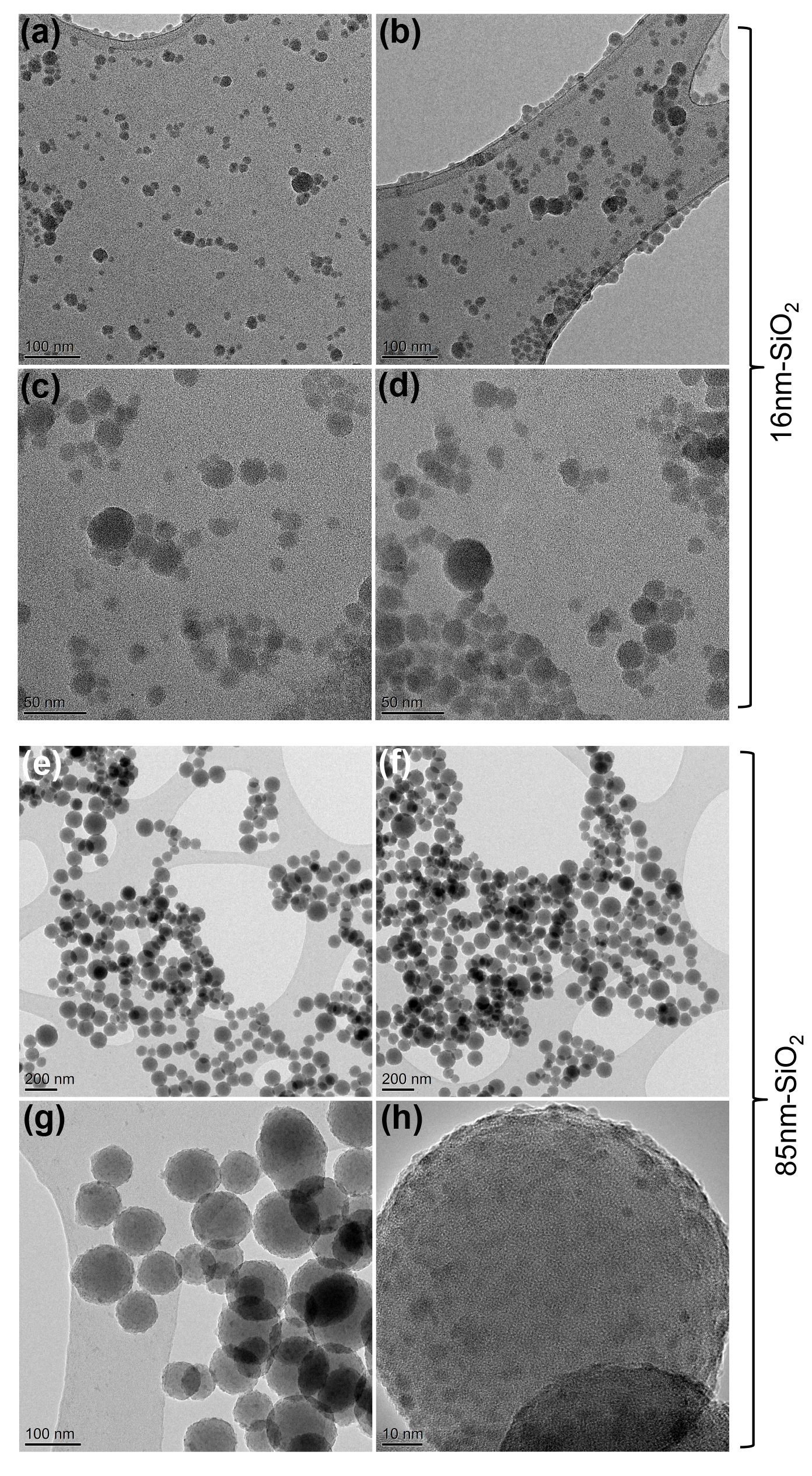

Supplement: Supplementary file 1 — Brightfield TEM micrographs of the BASF Levasil® silicon dioxide nanoparticles: 16 nm-SiO2 (a - d), 85 nm-SiO2 (e - h). (TIF 4314 kb) [file 12989_2016_161_MOESM1_ESM.tif]

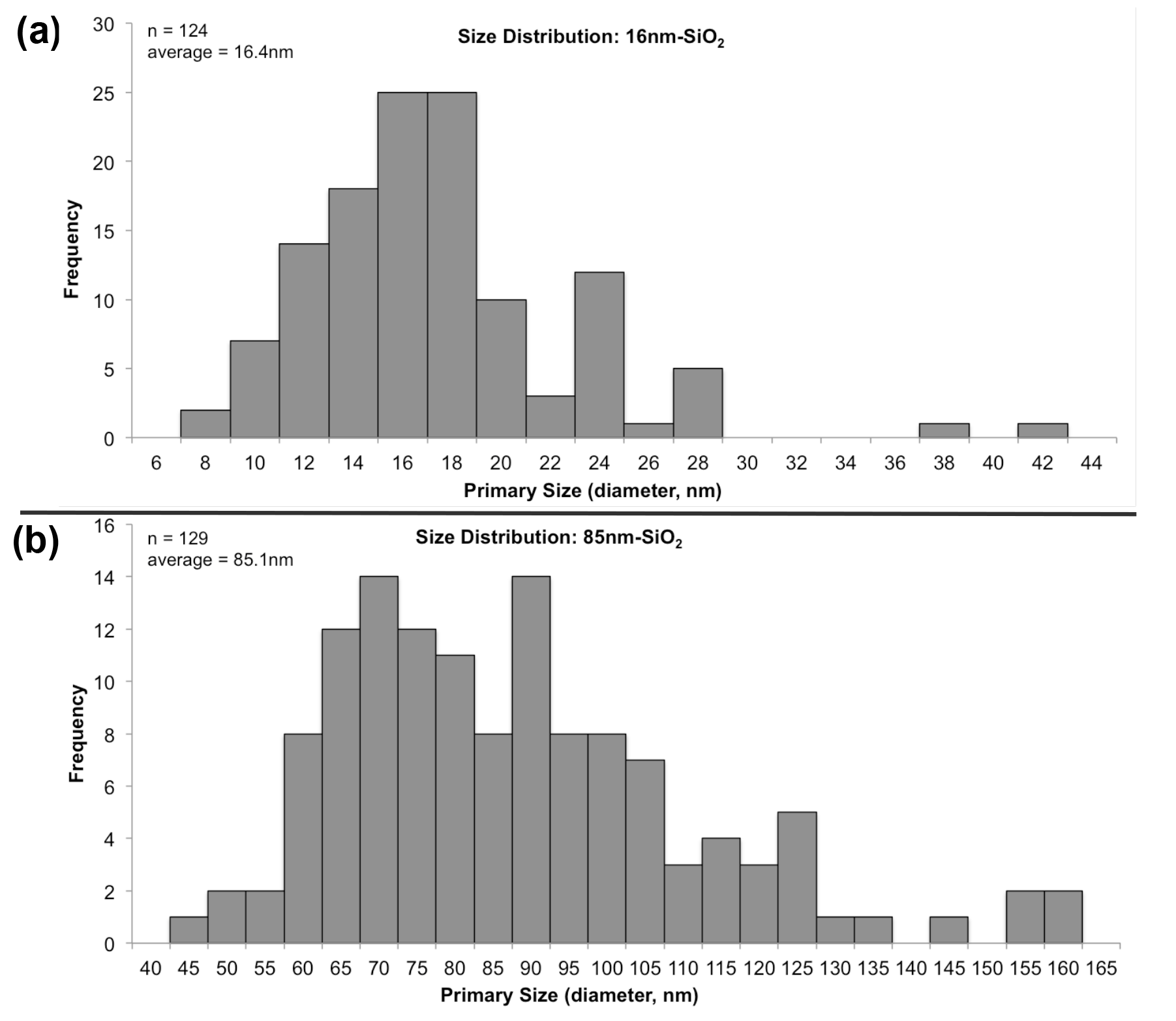

Supplement: Supplementary file 2 — Primary Levasil® size distributions calculated from TEM micrographs: 16 nm-SiO2 (a), 85 nm-SiO2 (b). (TIF 276 kb) [file 12989_2016_161_MOESM2_ESM.tif]

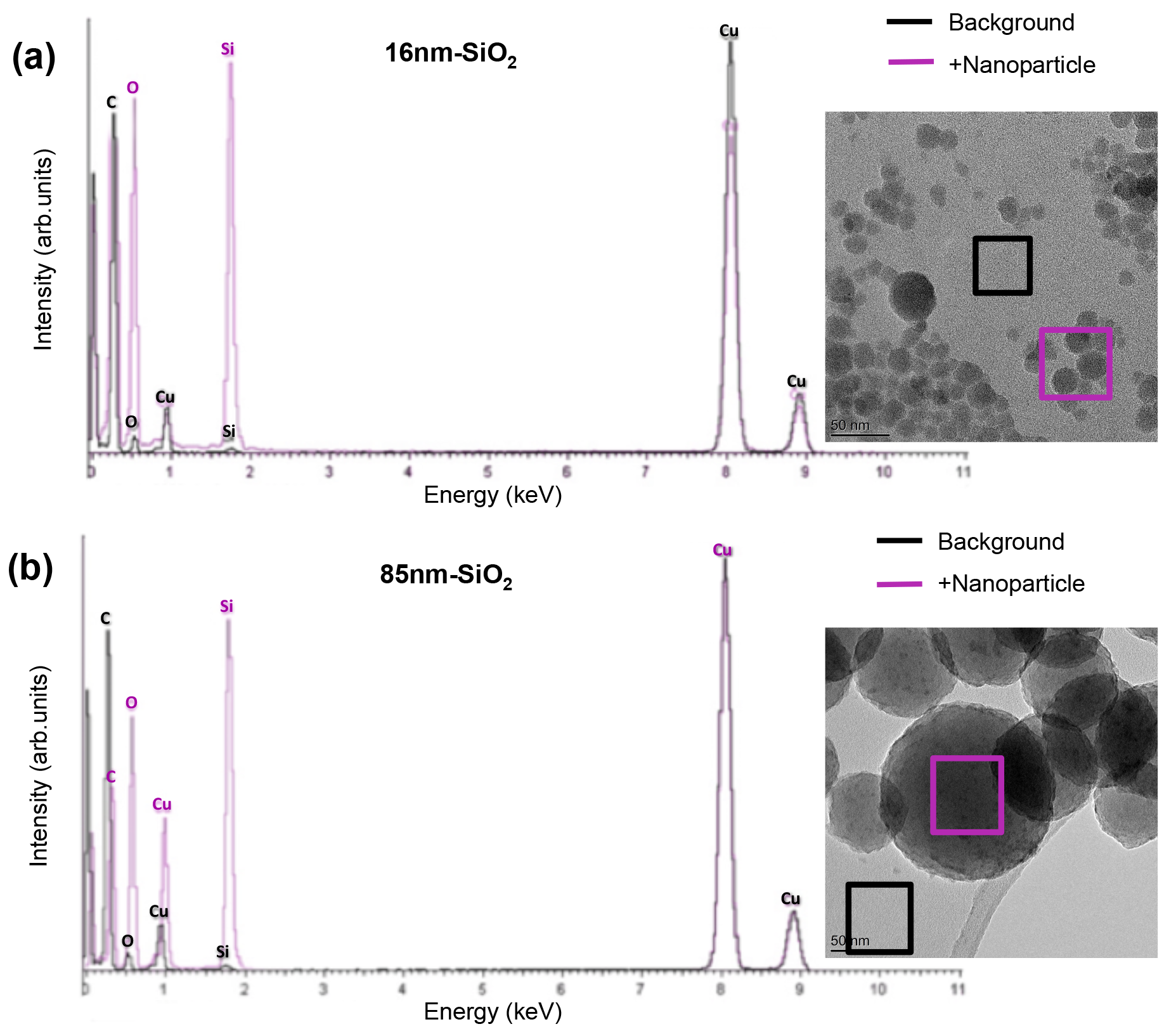

Supplement: Supplementary file 3 — Levasil® EDX spectra relative to background: 16 nm-SiO2 (a), 85 nm-SiO2 (b). Regions analysed shown in the inset image. Copper and carbon signals originate from the TEM grid and its support film. (TIF 1453 kb) [file 12989_2016_161_MOESM3_ESM.tif]

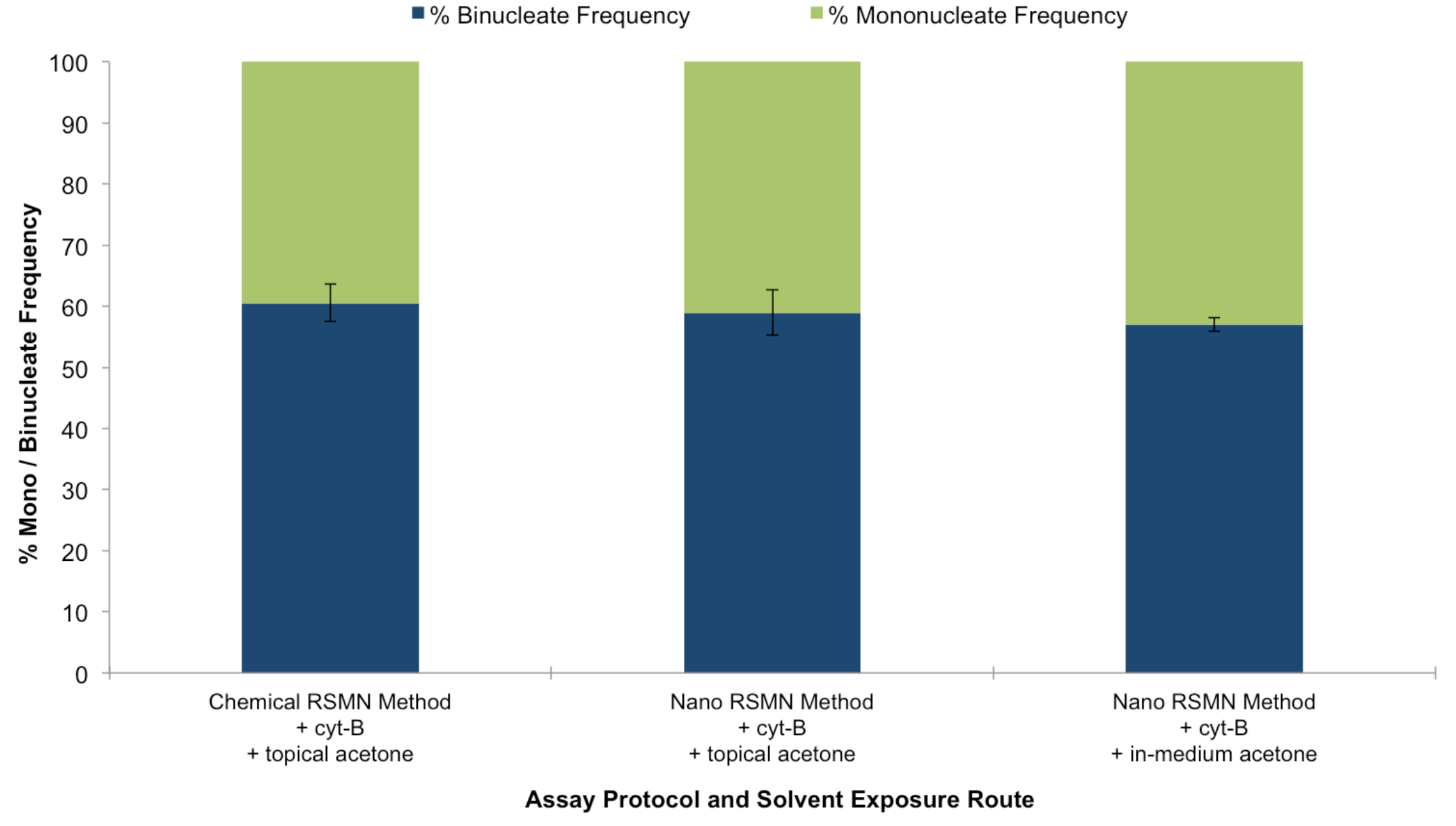

Supplement: Supplementary file 4 — Binucleate frequency comparisons between different 3D RSMN methods, cyt B regimes and solvent (acetone) inoculation routes: The optimised nano RSMN protocol designed to permit topical or in-medium nanoparticle exposures yielded highly similar frequencies of binucleated cells to the original protocol designed for chemical test articles (n = 3, error bars = SD). (TIF 218 kb) [file 12989_2016_161_MOESM4_ESM.tif]

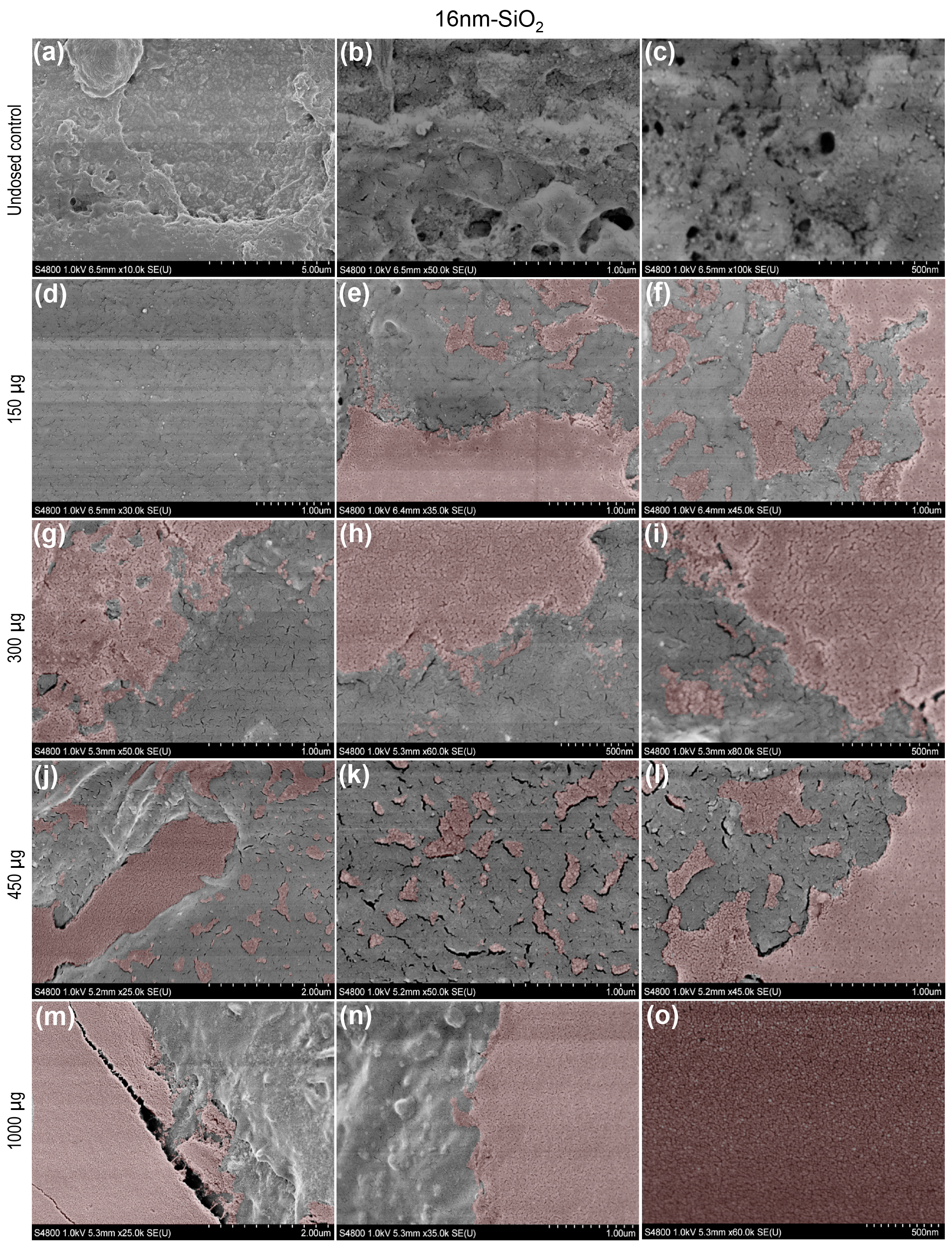

Supplement: Supplementary file 5 — Characterising 16 nm-SiO2 (false coloured red) topical 3D model deposition in acetone using cryogenic freezing and scanning electron microscopy: Dose increases down rows; images presented from separate samples prepared in triplicate. Alternative dose metrics including the 3D equivalent total mass doses with area (topical exposures) unit components are provided in Table 2. (TIF 7845 kb) [file 12989_2016_161_MOESM5_ESM.tif]

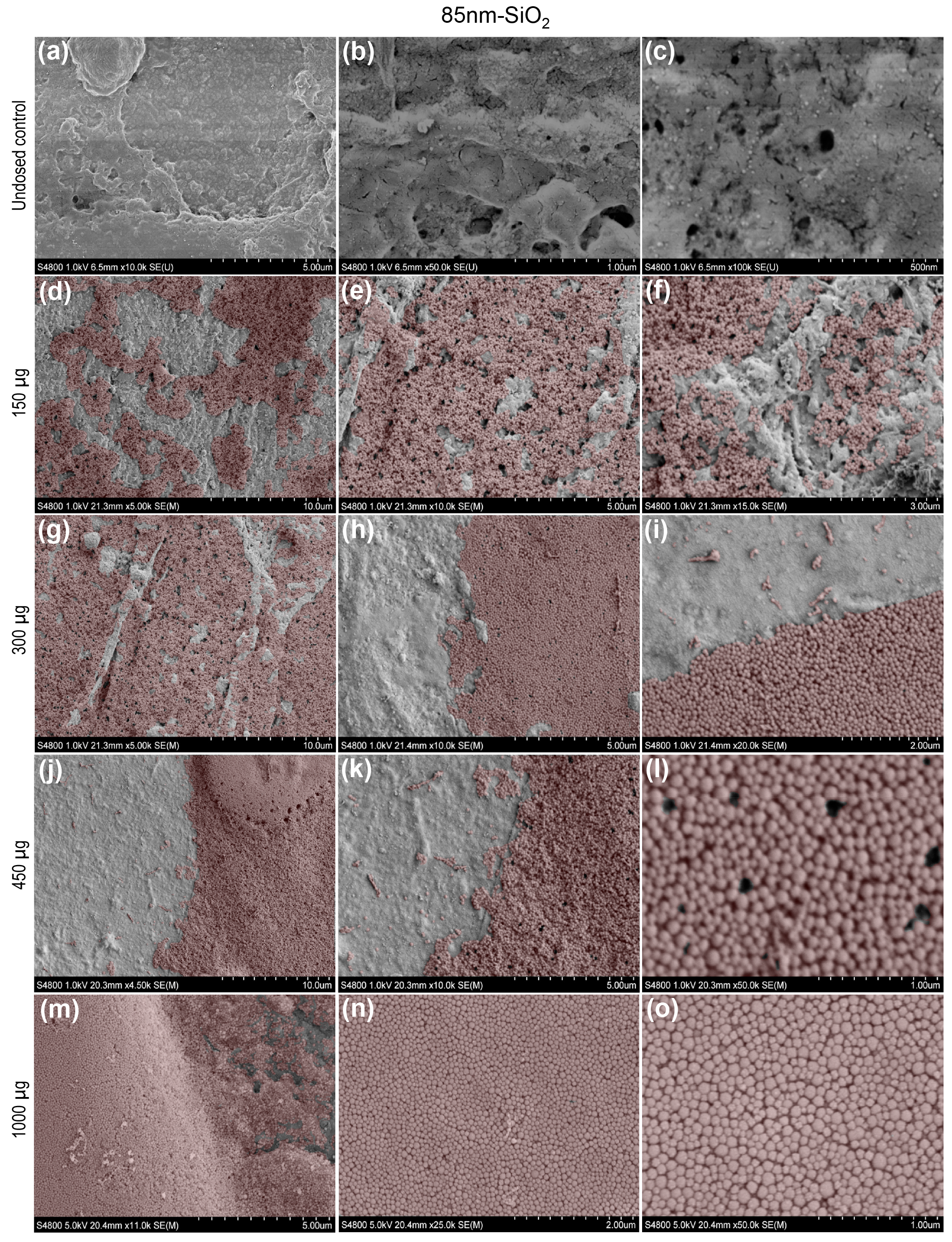

Supplement: Supplementary file 6 — Characterising 85 nm-SiO2 (false coloured red) topical 3D model deposition in acetone using cryogenic freezing and scanning electron microscopy: Dose increases down rows; images presented from separate samples prepared in triplicate. Alternative dose metrics including the 3D equivalent total mass doses with area (topical exposures) unit components are provided in Table 2. (TIF 9112 kb) [file 12989_2016_161_MOESM6_ESM.tif]

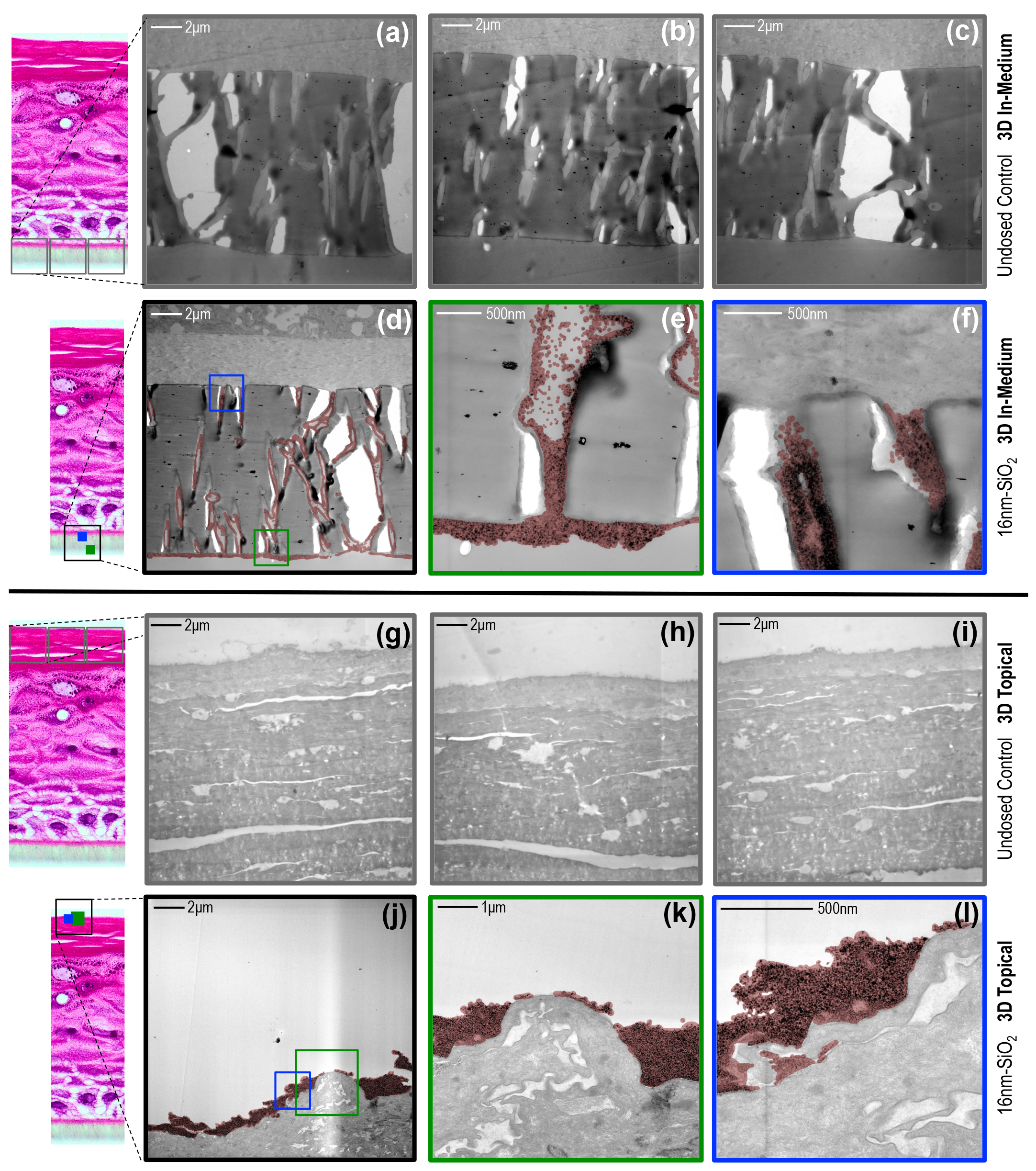

Supplement: Supplementary file 8 — Contrast inverted HAADF-STEM cross-sectional micrographs showing 16 nm-SiO2 nanoparticle localisation (indicated, red outline) in the 3D skin model: Images for each exposure route (450 μg exposures) are presented alongside undosed negative controls at the harvest time-point (+66 h) (images presented in triplicate by row). The inset light micrographs (left) show the position of the electron micrographs/particles in context of the complete tissue cross-section. Magnified regions (green/blue outlines) are indicated on the low magnification images (left column, black outlines) where applicable. Alternative dose metrics including the 3D equivalent total mass doses with area (topical exposures) and volume (in-medium exposures) unit components are provided in Table 2. (TIF 5694 kb) [file 12989_2016_161_MOESM8_ESM.tif]

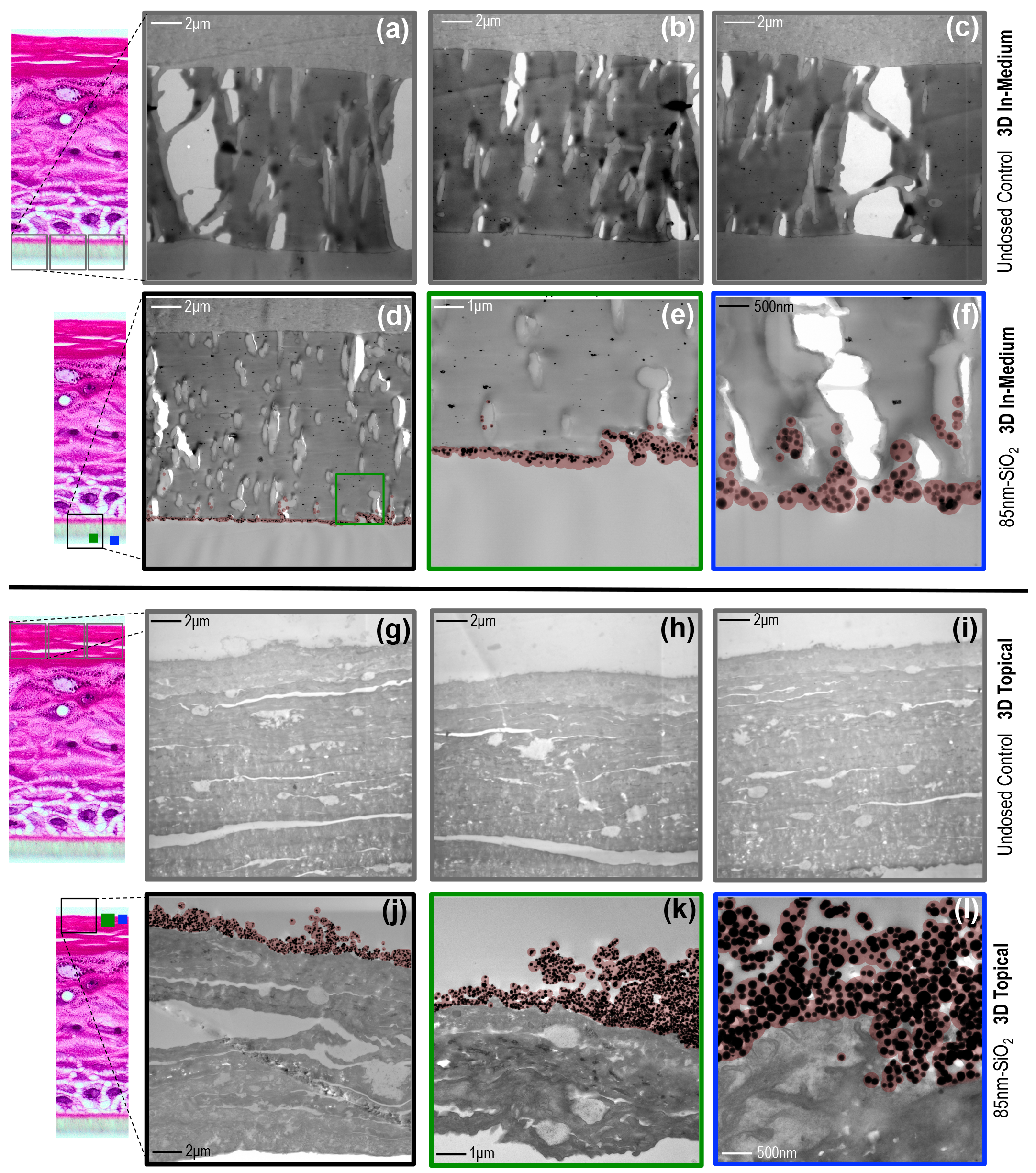

Supplement: Supplementary file 9 — Contrast inverted HAADF-STEM cross-sectional micrographs showing 85 nm-SiO2 nanoparticle localisation (indicated, red outline) in the 3D skin model: Images for each exposure route (450 μg exposures) are presented alongside undosed negative controls at the harvest time-point (+66 h) (images presented in triplicate by row). The inset light micrographs (left) show the position of the electron micrographs/particles in context of the complete tissue cross-section. Magnified regions (green/blue outlines) are indicated on the low magnification images (left column, black outlines) where applicable. Alternative dose metrics including the 3D equivalent total mass doses with area (topical exposures) and volume (in-medium exposures) unit components are provided in Table 2. (TIF 5412 kb) [file 12989_2016_161_MOESM9_ESM.tif]

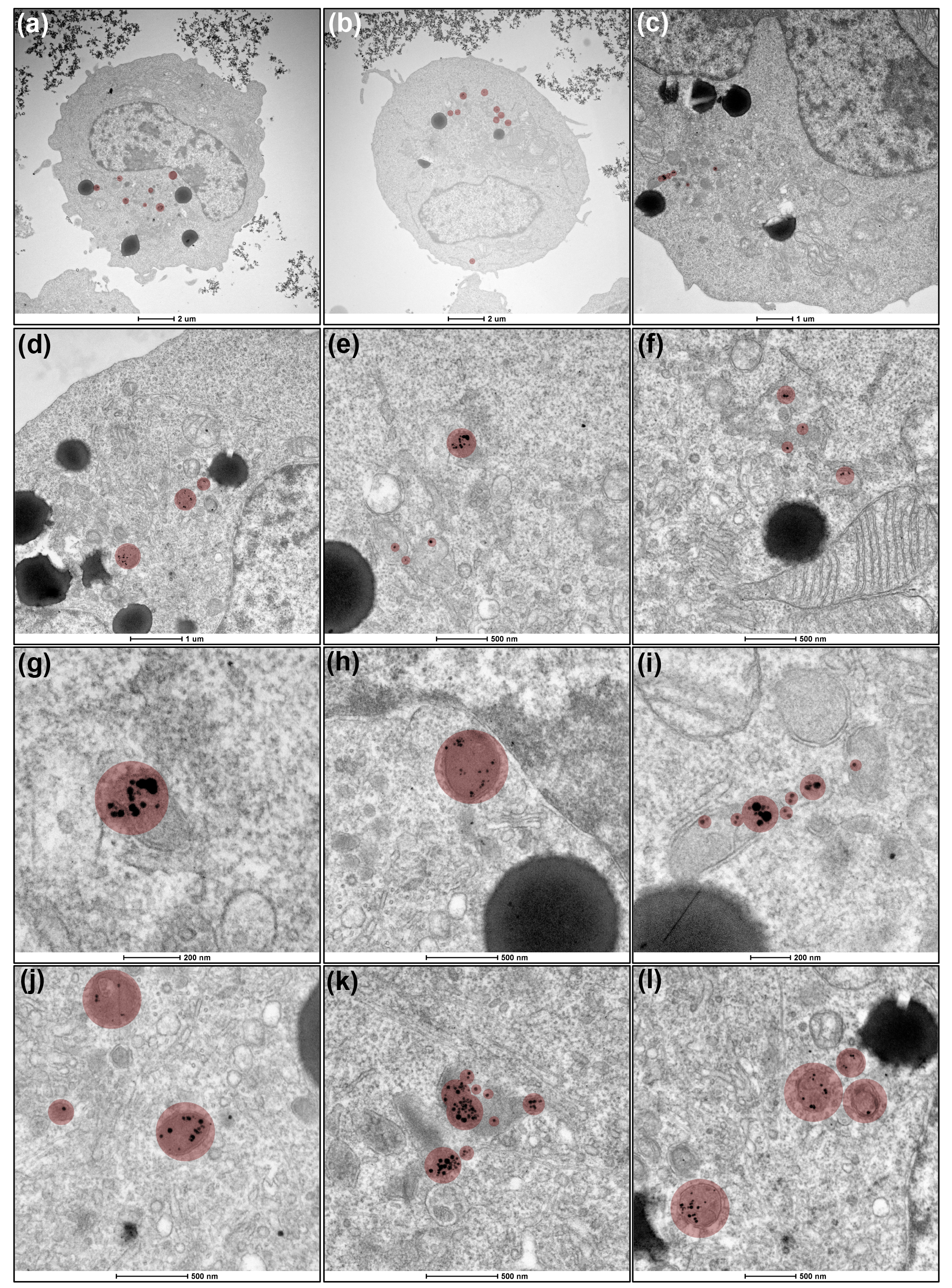

Supplement: Supplementary file 10 — Contrast inverted HAADF-STEM cross-sectional micrographs showing 16 nm-SiO2 nanoparticle uptake (indicated, red outline) in 2D TK6 lymphoblastoid cells: Images were taken at the harvest time-point (+42 h) (300 μg/mL dose). Efforts were made to image >50 cell sections. Particles could be seen surrounding / bound to cell membranes (a – c, black) and where readily internalised (red outlines) within vesicles (d – l) suggesting active uptake. Particle composition/uptake was confirmed by EDX spectroscopy. (TIF 7535 kb) [file 12989_2016_161_MOESM10_ESM.tif]

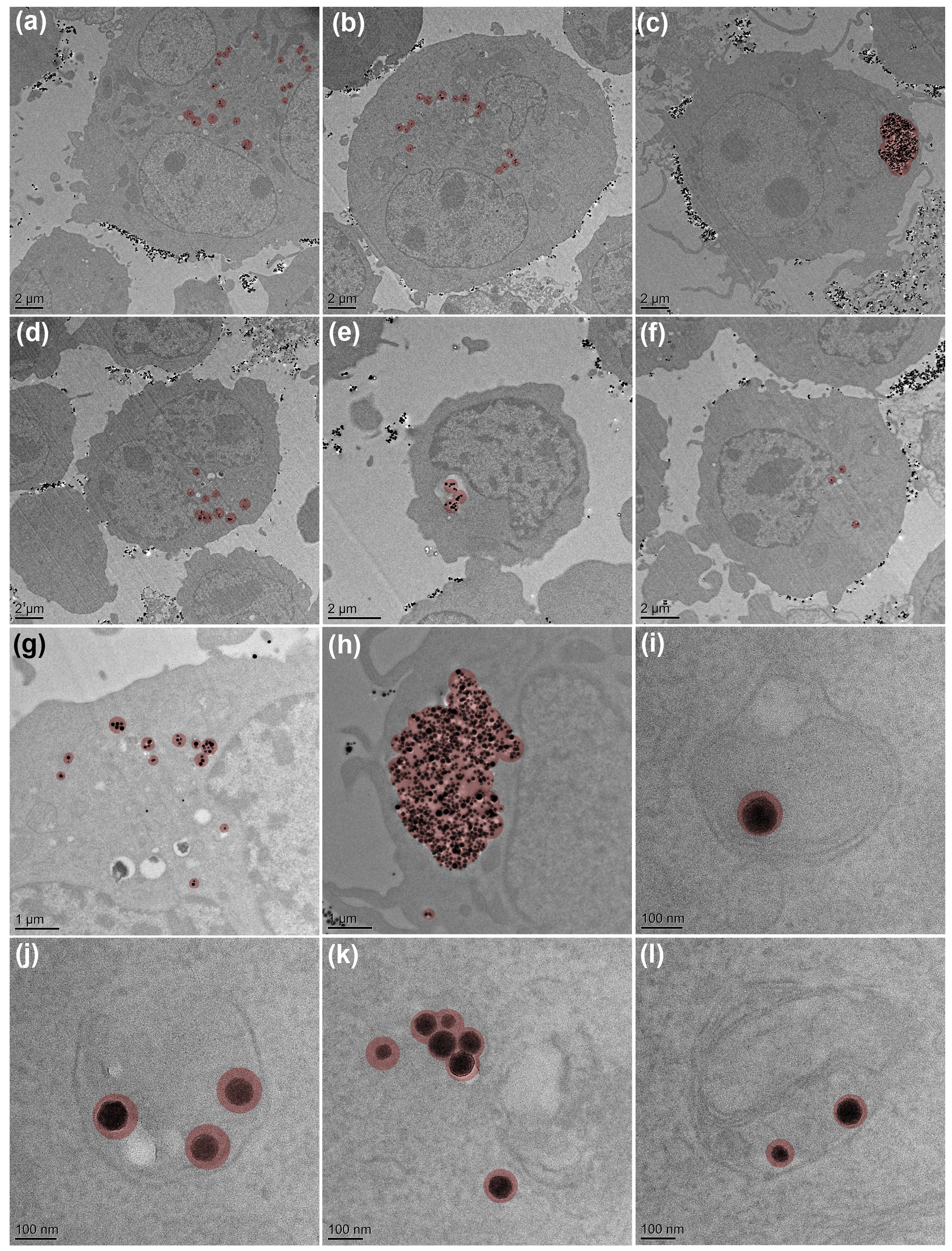

Supplement: Supplementary file 11 — Bright field TEM cross-sectional micrographs showing 85 nm-SiO2 nanoparticle uptake (indicated, red outline) in 2D TK6 lymphoblastoid cells: Images were taken at the harvest time-point (+42 h) (300 μg/mL dose). Efforts were made to image >50 cell sections. Particles could be seen surrounding / bound to cell membranes (a – f, black) and where readily internalised (red outlines) within vesicles (g – l) suggesting active uptake. Particle composition/uptake was confirmed by EDX spectroscopy. (TIF 8426 kb) [file 12989_2016_161_MOESM11_ESM.tif]

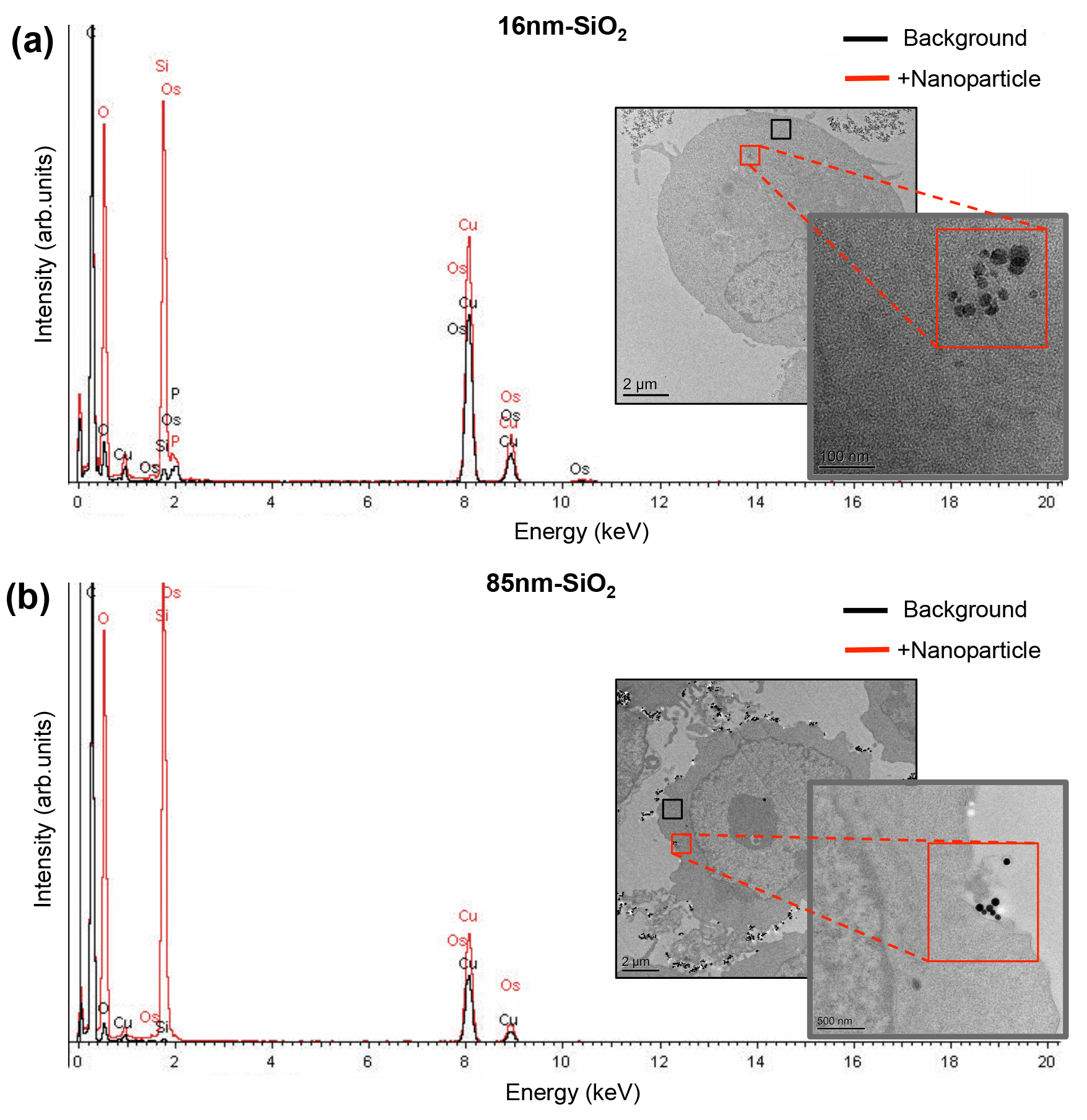

Supplement: Supplementary file 12 — Confirming 2D Levasil® uptake using EDX spectroscopy: (a) 16 nm-SiO2, and (b) 85 nm-SiO2. Areas analysed are shown in the inset images, with resulting spectra overlaid to compare background regions (black) to those containing nanoparticles (red). The copper signal originates from the TEM grid, and the carbon and osmium signals from the sample preparation method (e.g., the osmium tetroxide fixative used to enhance contrast and the resin used for embedding). (TIF 1653 kb) [file 12989_2016_161_MOESM12_ESM.tif]
